# Maintained imbalance of triglycerides, apolipoproteins, energy metabolites and cytokines in long-term COVID-19 syndrome patients

**DOI:** 10.3389/fimmu.2023.1144224

**Published:** 2023-05-09

**Authors:** Georgy Berezhnoy, Rosi Bissinger, Anna Liu, Claire Cannet, Hartmut Schäfer, Katharina Kienzle, Michael Bitzer, Helene Häberle, Siri Göpel, Christoph Trautwein, Yogesh Singh

**Affiliations:** ^1^ Werner Siemens Imaging Center, Department of Preclinical Imaging and Radiopharmacy, University of Tübingen, Tübingen, Germany; ^2^ Division of Endocrinology, Diabetology and Nephrology, Department of Internal Medicine I, University Hospital Tübingen, Tübingen, Germany; ^3^ Research Institute of Women’s Health, University of Tübingen, Tübingen, Germany; ^4^ Bruker BioSpin, Applied Industrial and Clinical Division, Ettlingen, Germany; ^5^ Department of Internal Medicine I, University Hospital Tübingen, Tübingen, Germany; ^6^ Center for Personalized Medicine, University Hospital Tübingen, Tubingen, Germany; ^7^ Department of Anesthesiology and Intensive Care Medicine, University Hospital Tübingen, Tübingen, Germany; ^8^ Institute of Medical Genetics and Applied Genomics, University of Tübingen, Tübingen, Germany; ^9^ Next Generation Sequencing (NGS) Competence Center Tübingen (NCCT), University of Tübingen, Tübingen, Germany

**Keywords:** long COVID-19, Post-Acute COVID-19 syndrome (PACS), NMR, in-vitro diagnostics, quantitative, metabolomics, lipoproteins, inflammation

## Abstract

**Background:**

Deep metabolomic, proteomic and immunologic phenotyping of patients suffering from an infection with severe acute respiratory syndrome coronavirus 2 (SARS-CoV-2) have matched a wide diversity of clinical symptoms with potential biomarkers for coronavirus disease 2019 (COVID-19). Several studies have described the role of small as well as complex molecules such as metabolites, cytokines, chemokines and lipoproteins during infection and in recovered patients. In fact, after an acute SARS-CoV-2 viral infection almost 10-20% of patients experience persistent symptoms post 12 weeks of recovery defined as long-term COVID-19 syndrome (LTCS) or long post-acute COVID-19 syndrome (PACS). Emerging evidence revealed that a dysregulated immune system and persisting inflammation could be one of the key drivers of LTCS. However, how these biomolecules altogether govern pathophysiology is largely underexplored. Thus, a clear understanding of how these parameters within an integrated fashion could predict the disease course would help to stratify LTCS patients from acute COVID-19 or recovered patients. This could even allow to elucidation of a potential mechanistic role of these biomolecules during the disease course.

**Methods:**

This study comprised subjects with acute COVID-19 (n=7; longitudinal), LTCS (n=33), Recov (n=12), and no history of positive testing (n=73). ^1^H-NMR-based metabolomics with IVDr standard operating procedures verified and phenotyped all blood samples by quantifying 38 metabolites and 112 lipoprotein properties. Univariate and multivariate statistics identified NMR-based and cytokine changes.

**Results:**

Here, we report on an integrated analysis of serum/plasma by NMR spectroscopy and flow cytometry-based cytokines/chemokines quantification in LTCS patients. We identified that in LTCS patients lactate and pyruvate were significantly different from either healthy controls (HC) or acute COVID-19 patients. Subsequently, correlation analysis in LTCS group only among cytokines and amino acids revealed that histidine and glutamine were uniquely attributed mainly with pro-inflammatory cytokines. Of note, triglycerides and several lipoproteins (apolipoproteins Apo-A1 and A2) in LTCS patients demonstrate COVID-19-like alterations compared with HC. Interestingly, LTCS and acute COVID-19 samples were distinguished mostly by their phenylalanine, 3-hydroxybutyrate (3-HB) and glucose concentrations, illustrating an imbalanced energy metabolism. Most of the cytokines and chemokines were present at low levels in LTCS patients compared with HC except for IL-18 chemokine, which tended to be higher in LTCS patients.

**Conclusion:**

The identification of these persisting plasma metabolites, lipoprotein and inflammation alterations will help to better stratify LTCS patients from other diseases and could help to predict ongoing severity of LTCS patients.

## Graphical Abstract

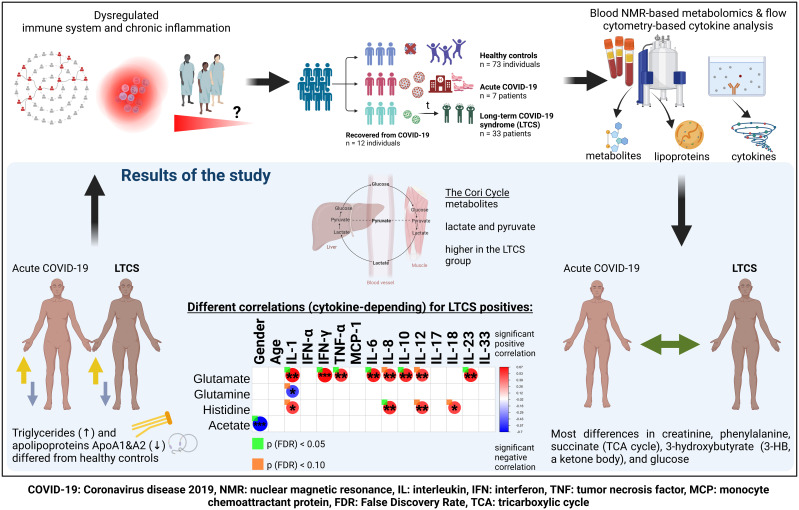

## Layman summary and significance of the research

Almost 10-20% of individuals infected with the SARS-CoV-2 virus regardless of hospitalization status experience long-term COVID-19 syndrome (LTCS). It is devasting for millions of individuals worldwide and hardly anything is known about why some people experience these symptoms even 3 to 12 months after the acute phase. Therefore, we attempted to understand whether dysregulated metabolism and inflammation could be contributing factors to the ongoing symptoms in LTCS patients. Total blood triglycerides and the Cory cycle metabolites (lactate and pyruvate) were significantly higher, lipoproteins (apolipoproteins Apo-A1 and A2) were drastically lower in LTCS patients compared with healthy controls. Correlation analysis revealed that either cytokines or gender are positively correlated with several metabolites (citrate, glutamate and histidine) in LTCS patients. Several cytokines and chemokines were also positively correlated with metabolites and lipoproteins thus, dysregulation in metabolism and inflammation could be a potential contributory factor for LTCS symptoms.

## Introduction

So far, more than 643 million people worldwide have been infected with COVID-19 and more than 6.6 million lives have been lost during the course of the pandemic (1). Yet, even three years after the first SARS-CoV-2 viral infections, the COVID-19 pandemic is still ongoing. Emergence of new variants of concern (VOC) is a great concern despite the development of several successful vaccines. Many scientific reports have identified the important role of metabolites in the serum and plasma of mild, moderate, severe, and recovered COVID-19 patients. In fact, in COVID-19 disease or any other viral infection, immune cells require a lot of energy to fight off the infection. Therefore, their metabolism demands a drastic increase to produce cytokines and chemokines (2, 3). A previous study described that peripheral blood mononuclear cells (PBMCs) show a dysregulated glycolysis and oxidative phosphorylation related metabolic profile, with specifically higher lactate and lower glucose levels in mild and moderate COVID-19 patients compared with either healthy controls (HC) or convalescent (Co) COVID-19 individuals (4). Furthermore, specific T cell subsets from acutely infected COVID-19 patients displayed a more extensive mitochondrial metabolic dysfunction, especially cells in CD8 T cell lineages (5). Finally, *in vitro* activated T cells from acutely infected COVID-19 patients showed a reduced glycolytic capacity and decreased glycolytic reserve, accompanied by a relatively low activation of mTOR signaling compared with HC (5). Of note, dysregulated metabolites can be released from both dysfunctional immune cells as well as from damaged tissue due to the viral infection in the blood (6, 7). Thus, the detection of metabolites from blood serum or plasma (reservoir and exchanger of metabolites) would give us a hint of the ongoing pathophysiological status of the disease in more detail.

Several studies have focused on how to predict and model the progression of COVID-19 based on metabolomics and proteomics, including the use of machine learning and mathematical modelling (7–13). These studies correlated metabolites with inflammation parameters and identified that alterations of several metabolites could be involved in disease progression, with some of them being a direct consequence of the disease. Further, in parallel considerable investigative efforts using genomics, transcriptomics and proteomics were performed on plasma and even fecal samples (14–20). A previous study by nuclear magnetic resonance (NMR) spectroscopy identified that lipoprotein subclasses and free cholesterol were increased in both mild and moderate COVID-19 patients, and this study concluded that COVID-19 causes a dysregulation in lipid metabolism, glycolysis, and the tricarboxylic acid cycle (21). Another NMR study of recovered COVID-19 patients (Recov) after 3–10 months of diagnosis indicated higher plasma cholesterol and phospholipids (22). Furthermore, changes in polar metabolites were determined, e.g. altered amino acids (arginine and glutamine were lower in COVID-19 patients (19)). Additionally, several studies highlighted that inflammatory cytokines such as IL-6 and IL-10 were present in highest levels in severe COVID-19 (acute) compared to moderate/mild or HC (21, 23, 24).

It is reported that several patients after infection develop a long term COVID-19 syndrome (LTCS) with symptoms such as chronic fatigue, dyspnea, brain fog, etc. (25). However, how COVID-19 specific metabolite, lipoprotein and inflammatory mediators relate to the severity of COVID-19 and LTCS outcomes remains poorly understood. Some studies suggested that mitochondrial dysfunction, impaired fatty acid metabolism and cytokine IL-10 production were greatly affected in LTCS patients (22, 26, 27). Thus, the role of host metabolism and inflammation during the disease progression in LTCS requires further investigation in defined patient cohorts including from different geographical regions to validate common and different features of this fatal health condition.

Of note, a previously launched *in vitro* diagnostics research (IVDr) NMR analytical platform demonstrated that for given samples this method can discover absolute quantitative data on metabolite and lipoprotein levels in analyzed solutions from either blood serum and plasma (28). This IVDr NMR platform has been already successfully implemented for COVID-19 phenotyping (12, 22, 29–36) and we have used the same platform for the purpose of this study.

The samples for this project were collected dated from June 2020 to February 2021 and correspond to the wildtype mutant of the virus based on epidemiological knowledge. In the current study, we aimed to perform similar investigations on LTCS and control cohorts using ^1^H-NMR based metabolomics, lipoproteome quantification and a targeted multiplex 13-plex inflammation panel. We hereby identified that the dynamics of metabolites, lipoproteins and inflammation parameters are altered in LTCS individuals.

## Materials and methods

### Study design and patient recruitments

We used four groups of individuals in this study. The four groups of participants included in this study (**Supplementary Table 1**) were defined as individuals with: Acute COVID-19 (n=7; with different time points - longitudinal) LTCS (n= 33); Recov (n= 12); and those who lacked any history of positive testing for COVID-19 (n= 73). A strict standardization in sample collection is crucial to obtain comparable results and samples were collected between 9 – 11 AM. in the morning under fasted conditions. Not all blood samples could be investigated by NMR and cytokine panel investigation, as not enough blood volume was available, or the cytokine panel failed. The n-numbers in **Supplementary Table 1** are therefore different for NMR and cytokines. Recov and LTCS groups were seen in an ambulatory clinical setting. HC samples (except the additional controls from Bruker BioSpin; see below) were recruited for normal blood donation and checked for IgG and IgM antibodies levels to make sure they had no previous SARS-CoV-2 infection (n= 32). Additional HC data (n=41) provided by Bruker BioSpin GmbH was generated prior the COVID-19 pandemics. The applied analytical approach by quantitative IVDr-NMR is a certified toolbox. In several studies it has shown that data from different times and research sites provide absolute comparability and reproducibility as the same platform, data generation and data processing algorithms are used. All participants enrolled were of at least 18 years of age. LTCS individuals presented patients evaluated at the Tübingen University Hospital for Post-COVID Care between June 2020 and February 2021 and part of a multi-omics study cohort (COVID-19 NGS; Ethics number: 286/2020B1 and Clinical Trial number: NCT04364828). They were enrolled only if blood was collected > 28 days after testing positive by SARS-CoV-2 PCR and were experiencing any symptoms such as fatigue, dyspnea, brain fog etc. The following additional metadata parameters were received and considered for analysis: age and gender status (0 – male, 1 – female, for the purpose of categorization within statistical software). This study was performed in accordance with the Declaration of Helsinki and all patients have been given written consent.

### Sample preparation for the study

Blood samples were collected in the morning at the clinics and delivered to our institute in the afternoon. Initial samples were collected in 9.0 mL EDTA tubes (S-Monovette^®^ K2 EDTA Gel, 9 ml, cap red; Sarstedt, Germany) for the isolation of DNA for genomic and epigenomic investigations. After completing routine blood tests in the clinical laboratory, the remaining discarded blood samples (2-5 mL) were used for plasma and PBMCs isolation to analyse metabolites, lipoproteome and inflammation parameters. Plasma separation was performed within 3-4 h after blood collection by centrifuging the blood samples at 2,000 x g for 10 min at room temperature and collected the upper layer. Plasma was stored at – 80°C or until use for both IVDr NMR spectroscopy and 13-plex inflammatory cytokine panel measurements.

### Flow cytometry-based 13-plex inflammatory cytokine assay

To determine cytokine levels from plasma samples obtained from HC, Recov and acute COVID-19 patients, we employed the LEGENDplex™ Human Inflammation Panel 1 (13-plex) flow cytometry-based assay kit (#740809, BioLegend, San Diego, CA, USA). This panel allowed us simultaneous quantification of 13 human inflammatory cytokines and chemokines (IL-1β, IFN-α2, IFN-γ, TNF-α, MCP-1 (CCL2), IL-6, IL-8 (CXCL8), IL-10, IL-12p70, IL-17A, IL-18, IL-23, and IL-33). The measurement principle is based on beads which are differentiated from each other based on their size and internal fluorescence intensities on a flow cytometer platform. Each bead set is bound with a specific antibody on its surface and forms capture beads for individual analytes. To detect the cytokine levels, we followed the protocol as recommended by manufacturer’s instruction. Briefly, we first prepared the standard using 1:4 dilution of the top standard (C7) as the highest concentration, then serial dilutions were done for C6, C5, C4, C3, C2, and C1 by taking 25 µL of the diluted standard and added into 75 µL assay buffer. Following, 15 µL of plasma samples were equally diluted with 15 µL assay buffer. Next, 25 µL of the diluted samples were carefully transferred to each well. 25 µL of mixed beads were added to each well. Importantly, beads were mixed well by vortex for 30 seconds before using to avoid bead setting in the bottle. The plate was sealed with a plate sealer and covered with aluminum foil to protect the plate from light and put on a plate shaker at 800 rpm for 2 h incubation at room temperature (RT). After incubation, the plate was centrifuged at 1.050 rpm for 5 minutes, then the supernatant was carefully discarded by flicking the plate in one continuous and forceful motion. The plate then was then washed with 200 µL washing buffer. 25 µL of detection antibodies were added to each well, the plate was again sealed with a plate sealer, covered with aluminum foil, and incubated for 1 h at RT. After incubation, 25 µL of streptavidin-phycoerythrin (SA-PE) was directly added to each well without washing the plate, sealed and covered in the same manner as described in a previous step. The plate was then centrifuged for 5 minutes and washed in the same manner as before. Finally, 150 µL of washing buffer was added to each well and the samples were stored in the cold room until the reading by BD Fortesa (BD Bioscience) flow cytometer.

Data were analyzed both manually and automatically by standard curve detection (online software platform from Biolegend). In automatic gating strategy, two sets of beads were used in this experiment. Each set has a unique size that was identified by its forward scatter (FSC) and side scatters (SSC) profiles. Based on the internal fluorescence intensities of each set of beads, different resolutions were achieved by flow cytometry. BD Fortesa flow cytometer was used for the internal dye detection *via* the APC channels. In Beads A there are six bead populations, whereas, in Beads B, there are seven bead populations. The predicted concentration of the cytokine standard levels was depicted in different colors. C7 represents the highest level of cytokines and C0 represents the lowest level of cytokines. Log5P analysis were performed to calculate the concentrations of each cytokine for multiple samples based on cloud-based online software provided by BioLegend.

### ^1^H-NMR spectroscopy-based metabolomics and lipoprotein quantification

Raw NMR spectra were recorded using Bruker IVDr (B.I.) methods package for blood samples, which is compatible with EDTA-(ethylenediaminetetraacetate), citrate-, and heparin blood plasma as well as serum samples (37). The sample preparation was performed following standard operating procedure (SOP) to ensure reliable results. For quality control, the B.I. BioBank QC™ module was applied. For quantification, the modules B.I. QUANT-PS™ for metabolites and B.I. LISA™ for lipoproteins, respectively, were applied. Blood plasma samples were thawed for approximately 30 minutes at RT. An aliquot of 120 μL of each aliquot was pipetted into a 1.5 mL polytetrafluoroethylene (PTFE) container and mixed with 120 μL of commercially prepared pH 7.4 sodium phosphate plasma buffer (Bruker BioSpin GmbH, Ettlingen, Germany). The mixture was then shaken gently for 1 min before transferring 200 μL of it to fill a 3 mm NMR tube (Bruker BioSpin GmbH, Ettlingen, Germany). The autosampler cooling setting was set to 4°C. 1D ^1^H-NMR spectra were acquired using a 5 mm triple resonance (TXI; ^1^H, ^13^C, and ^15^N) RT probe on a Bruker IVDr Avance III HD 600 MHz system (Bruker BioSpin GmbH, Ettlingen, Germany), which was operated using Bruker’s standard NMR software TopSpin (version 3.6.2). Five one-dimensional ^1^H-NMR spectral experiments were run for each blood sample with water peak suppression and varied pulse sequences to selectively observe molecular components. Firstly, a Nuclear Overhauser Effect SpectroscopY (NOESY) 32-scan NMR experiment was used to show NMR spectrum quality (via the B.I. BioBank QC™) and to enable quantification of metabolites (e.g. glucose, lactic acid, amino acids of the B.I. BioBank Quant-PS™) and high-molecular-weight compounds lipoproteins (as shown in B.I. LISA™). Then, a 32-scan (CPMG Carr-Purcell-Meiboom-Gill, filtering out macromolecular resonance signals) program was run, as well as 32-scan DIFFusion measurement of, primarily, macromolecular signal massifs (DIFF). Also, a two-dimensional NMR experiment is included within the IVDr methods package and 2-scans J-RESolved spectroscopy (JRES) were recorded to analyse J coupling constants. Additionally, JRES can be useful for a manual data look-up. NMR experiments utilize a group of sample-dependent parameters of frequency offset O1 and duration of 90° pulse P1. Using the B.I. QUANT-PS™ module, final concentration values as per reports were used for analysis. The annotation and quantification of serum spectra were provided automatically and server-based by Bruker BioSpin GmbH. Herein, 38 metabolites (via Bruker IVDr Quantification in Plasma/Serum, B.I. Quant-PS™, analysis package) and 112 lipoprotein parameters (via Bruker IVDr Lipoprotein Subclass Analysis, B.I. LISA™, analysis package; **Supplementary Table 2**) were identified and quantified in all spectra. As input, final concentrations from B.I. reports were employed.

### Statistical analysis

Statistical analysis was performed with the quantified parameters using the web-based tool MetaboAnalyst 5.0 (38). For the software’s analyses, we excluded all features that showed >50% missing values, metabolite and cytokine data panels only. Importantly, the over 50% missing value threshold is already stringent for more than three or more than three group-based comparisons, and these comparisons are functioning per feature. The remaining missing values were estimated (imputation) using the feature-wise replacement with 1/5 of a minimum variable value *via* the Singular Value Decomposition (SVD) computation (39). Different to lipoproteins, blood serum metabolites are amenable to fall below the analytical limit of quantification which is dependent on the individual metabolite SNR (signal to noise ratio). In order to use such metabolites still for statistics, we applied an imputation method accounting for up to 50% of missing values, as within disease research a single group (e.g. LCTS) might show distinct unique features that are not measured in any of the other groups (e.g. HC).

The probabilistic quotient normalization (PQN) technique was used to adjust for dilution effects in the corresponding metabolite concentration spreadsheets (40). To correct for heteroskedasticity, which is not uncommon in this context as concentration magnitudes from metabolites, lipoproteins, and other markers vary strongly, we performed a logarithmic transformation prior to statistical analysis. For univariate analysis, volcano plots were generated (combination of p-values generated from unpaired t-tests, and fold change (FC)). For figure generation, thresholds for the p-value were established at 0.10 and for the FC at 1.2, respectively. However, for two-group based comparisons (with no normalization techniques applied; unequal group variance; non-parametric t testing) were carried out *via* the utilization of Wilcoxon Rank Test, including false discovery rate (FDR) adjustment for output p values. For correlation analyses, including PatternSearch function of MetaboAnalyst, we focused on Spearman’s correlation coefficient. Further analyses were conducted using the multivariate approach of unsupervised principal component analysis (PCA) and supervised orthogonal partial least squares discriminant analysis (oPLS-DA). Besides that, PLS-DA was used to assess the discrimination between two groups and identify the parameters that drive this separation. MetaboAnalyst’s biomarker toolbox was used for further biomarker analysis (41). Without any alteration to the data matrix, such as logarithmic scaling or elimination of zero values, the pathway analysis tool worked correctly with only metabolite data. The univariate analysis (via Mann-Whitney tests), correlational analysis were performed and violin plots were illustrated using GraphPad PRISM 9.0.1. However, the main correlational analysis of combined NMR and cytokine data (without any alteration to the data matrix, such as logarithmic scaling or elimination of zero values; including FDR-method adjusted p-value calculations) was conducted using the “bcdstats” R package. BioRender.com services were utilized to create some figures within this work.

## Results

### Cohort description and patient demographics

**Figure 1:**
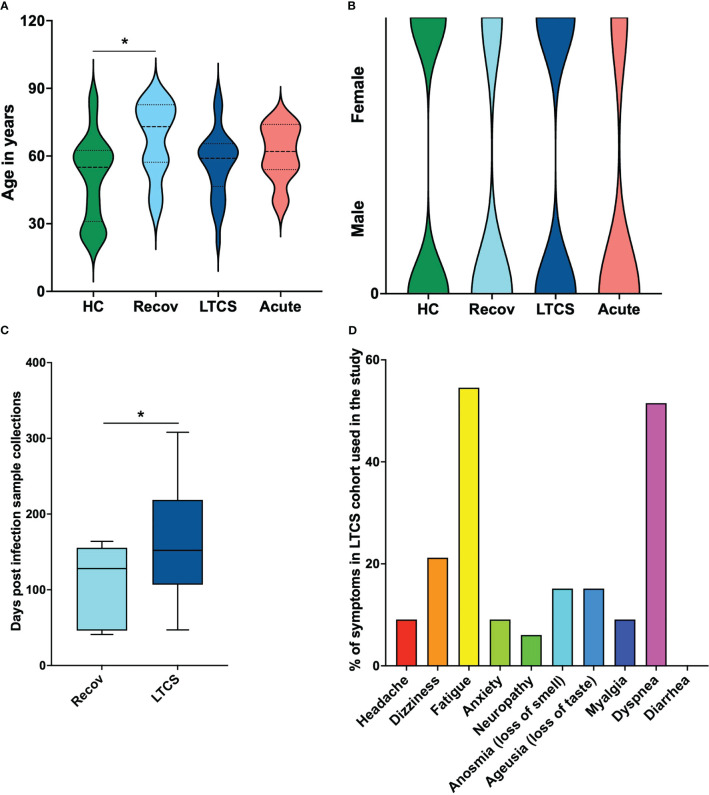
Patient demographics of LTCS patients and study cohort. **(A)** Age dependency in clinical patient groups and a substantial age difference between HC and recovered patients. **(B)** Gender-based structural map for the patient groups. **(C)** Sample collection time. There is a statistically significant difference between the recovered group and the LTCS group in number of days post infection registered. **(D)** The rows graph shows percentages, comorbidities as subclinical cofactors for the LTCS group. A statistically significant difference was indicated when the value of P was less than 0.05 (* - P ≤ 0.05).

To better interpret the obtained NMR and cytokine data, basic metadata from all recruited patients was considered in this study. We hereby identified in the healthy control group (HC) an age average of 54.4 years, whereas Recov was 67.5 years, LTCS was 56.9 years and acute COVID-19 was 61.1 years (**Figure 1A**). Kruskal-Wallis multiple test comparison revealed that the HC group age was significantly less compared with Recov (p_adj_=0.022, Dunn’s multiple comparisons test multiplicity adjustments performed) patients. However, no statistical difference was observed among Recov, LTCS, and acute COVID-19 patients’ age. Gender based analyses were also performed for each group and male and female subjects appeared to be distributed equally in HC and LTCS patients (**Figure 1B**; **Supplementary Table 1**). Further, we identified the post-acute COVID-19 (more than 4 weeks) or LTCS patient sample collection from date of infection to plasma collection for the study based on NICE guideline released in November 2020 (42). Later 7^th^ December 2022, World Health Organization (WHO) defined LTCS as the continuation or development of new symptoms 12 weeks after the initial SARS-CoV-2 infection, with these symptoms lasting for at least further 8 weeks with no other explanation. We identified that the median of the sample collection was 152 days which qualify the NICE and WHO guidelines. In our cohort, we had few samples (<10% patients (n=3: 47, 73, 80 days) used in this study) after viral infection (wild-type SARS-CoV-2) with minimum 47 and maximum 308 days (**Figure 1C**), whereas in the case of Recov patient group it was 128 days (**Figure 1C**). LTCS and Recov patients sample collection was significantly different (p=0.03), thus it appeared that LTCS patients and Recov patients had a clear demarcation of the symptoms. Herein, a total of ten major different symptom parameters was used to define LTCS patients: we identified that our cohort (n=33) had fatigue (>54.5%), dyspnea (>51.5%), dizziness (>21%) as major symptoms (**Figure 1D**). Anosmia (>15%), ageusia (>15%), headache (>9%), anxiety (>9%), myalgia (>9%), and neuropathy were fewer common symptoms (>6%).

### Dysregulated metabolites in severe and LTCS patients

**Figure 2:**
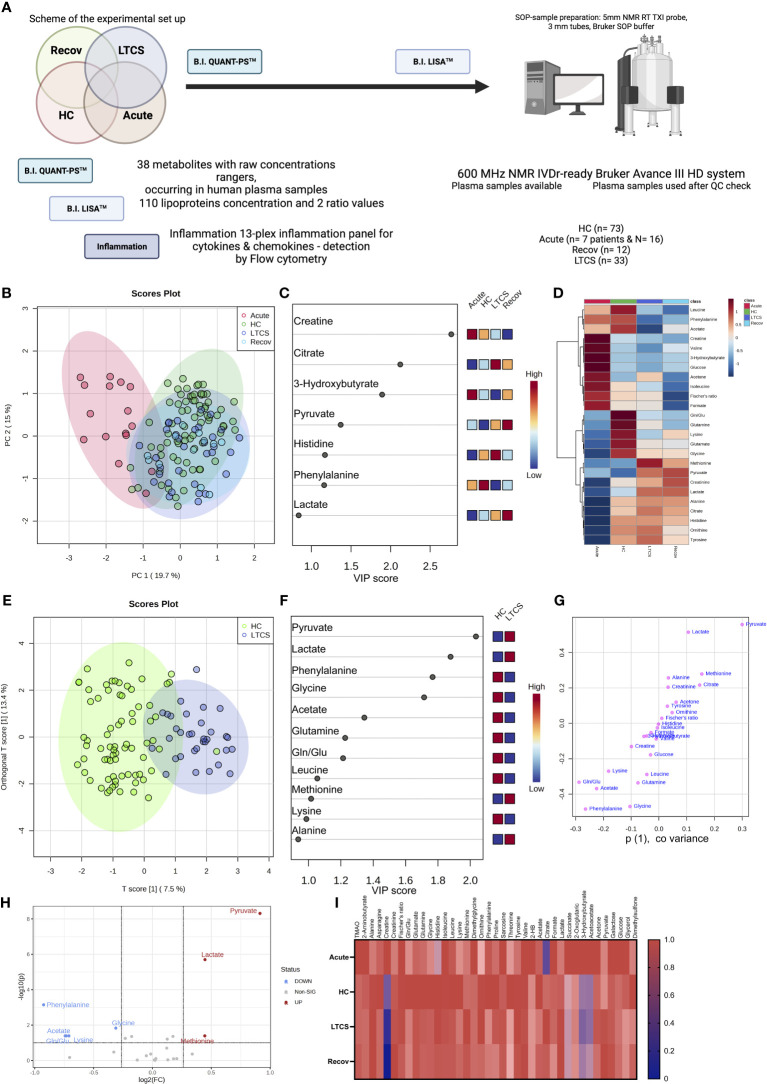
Identification of metabolites in LTCS patients. **(A)** The image above depicts the study’s methodology. **(B)** PCA and PLS-DA studies were performed for the whole cohort data. This analysis was done out based only on quantifiable metabolites data (B.I. QUANT-PS™). The 4-group distribution was shown in using the coordinates of principal components 1 and 2. **(C)** The values that contributed the most to these VIP scores are shown here by the subplot, which are sorted from most significant to least significant. **(D)** The metabolite panel variables’ average trends were presented by sub-plot. **(E)** oPLS-DA study was performed, and LTCS vs control patients (HC) were compared. This analysis was done out based only on quantifiable metabolites data (B.I. QUANT-PS™). The two-group distribution was shown using the coordinates of loading components 1 and 2. **(F, G)** The values that contributed the most to these VIP scores and S-plot data of the regression model are shown here by the subplots, which are sorted from most significant to least significant. **(H)** Metabolite panel Volcano analysis results showing trend-like (FC > 1.2, p (FDR-adjusted) ≤ 0.10) changes in ratio of LTCS/HC as presented by sub-plot. **(I)** For each patient group (Recov (EDTA plasma) n=12, HC (serum or heparin plasma) n=73, LTCS (EDTA plasma) n=33, Acute (Heparin plasma) n=16 samples), an average normalized (scaled 0 to 1, averages were divided by a maximal average per variable) heat map analysis conducted by sub-plot.

Several studies identified that blood metabolites are dysregulated especially in severe COVID-19 patients and in recovered patients (3, 41, 43–45). This is further affected by different variant strains and collection times (46). However, information on how metabolites and inflammation parameters affect LTCS patients has started to emerge only recently (22, 26). In our study, we used quantitative IVDr ^1^H-NMR spectroscopy to distinguish metabolites levels in HC, Recov, LTCS, and acute COVID-19 patients. The herein applied IVDr metabolomics biofluid approach was first introduced in 2016 (47) and is based on harmonized SOPs for sample preparation and data acquisition by (^1^H) 600 MHz NMR. In order to validate the reproducibility of the IVDr NMR methods, different ring trials were performed by both the analytical company as well as by the research community (34). We first compared the entire cohort of samples with different groups based on quantifiable metabolite data (B.I. QUANT-PS™) and untargeted PCA. Later on, there will be a section about the 112 parameters (B.I. LISA™) available for investigation on the lipoprotein panel data (**Supplementary Table 2**). We hereby found that the acute COVID-19 patient group showed a clear separation with either LTCS, Recov or the HC group (**Figure 2B**). PCA loading scores investigation was performed as well (**Supplementary Figure 1**). Further, PLS-DA’s variables in projection importance score plot (VIP) suggested that the amino acid creatine and the ketone body 3-hydroxybutyrate was present at the highest level in the plasma samples of acute COVID-19 patient whilst citrate and histidine were present in LTCS patients at highest levels among all other groups (**Figure 2C**). Recov patients showed the highest amount of pyruvate and lactate levels (**Figures 2C, D**), as illustrated also on the heat map plot. Further, we identified that formate, acetone, and citrate were present in higher amounts in LTCS compared with Recov patients (**Supplementary Figure 2**). Due to the limited number of samples in the Recov and acute group, we mostly focused for this study on the comparison between HC and LTCS. A supervised classification model was built using oPLS-DA to distinguish between HC and LTCS patients, using metabolites as variables. We observed a clear difference and elevated levels of pyruvate, lactate, methionine and alanine in LTCS patient compared to HC (**Figures 2E-G**). The regression analysis also highlighted that pyruvate, lactate and methionine as top variables in the oPLS-DA S-plot. Furthermore, the metabolite panel in the volcano analysis showed trend-like (FC > 1.2, p (FDR-adjusted) ≤ 0.10) changes for lactate, pyruvate, and methionine (up) and phenylalanine, glycine, Gln/Glu (glutamine-glutamate ratio), lysine and acetate (down) in LTCS compared with HC (**Figure 2H**). Finally, we compared and revealed the overall changes in the metabolites among all different groups (**Figure 2I**).

We delineated that LTCS compared with acute COVID-19 patients have a highly significant change in several metabolites including alanine, histidine, citrate, lactate, pyruvate, and glucose (**Supplementary Table 3**, FDR-adjusted significances shown). Yet, we have been unable to establish any differences between the LTCS and Recov groups that are statistically significant. This is also not surprising, as the n-number for the Recov group is very small. The examination by a regression model, however, made it possible to identify several indicative changes (**Supplementary Figure 2**). Those were elevated levels of formate in LTCS, but the group also had a tendency of lowered amounts of acetate, creatinine, lysine, valine, pyruvate, phenylalanine, and lactate when compared with Recov individuals. Overall, the energy metabolites of citrate and pyruvate were much higher in the LTCS and Recov groups than in acute COVID-19 patients (**Figure 2**; **Supplementary Figure 3**).

We next identified pathway alterations. Overall, six pathways were mainly identified which had a significant difference including the TCA cycle, ketone bodies, alanine/aspartate/glutamate metabolism, glycolysis, glycine/serine/threonine metabolism, and arginine/proline metabolism. From all six pathways, metabolites from the glycolysis pathway were deemed to be less abundant in acute patient samples. At the same time, TCA cycle metabolites were high in both Recov and LTCS patient groups with high significance levels. Finally, we were able to observe slightly lowered levels of glycolysis metabolites in the LTCS group as well. Thus, our data defines a metabolic dysregulation in LTCS and acute COVID-19 patients.

### Imbalanced lipoproteins are key characteristics for LTCS

**Figure 3:**
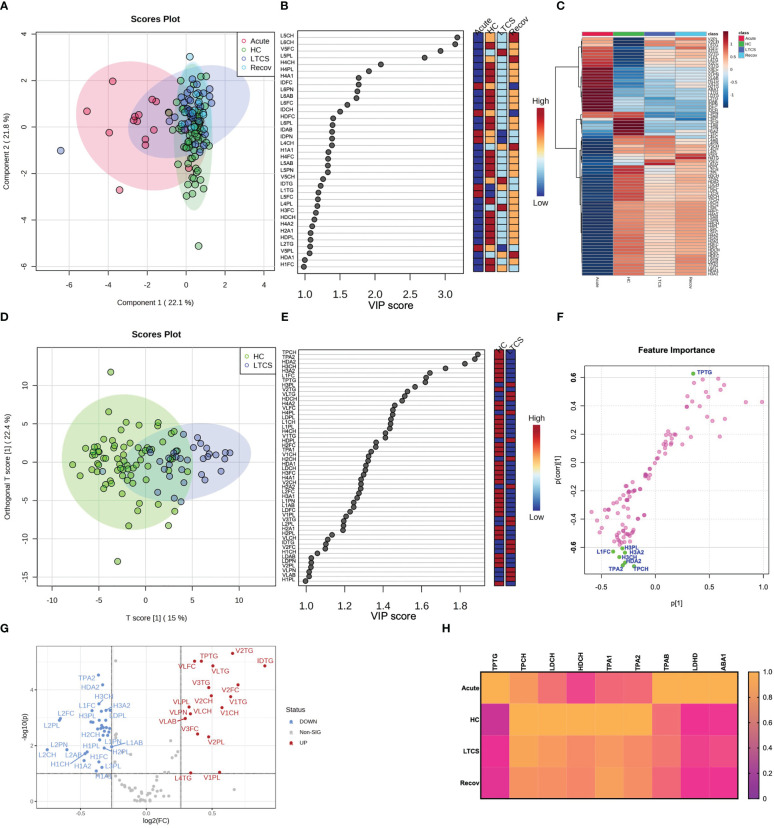
Lipoprotein profiling in LTCS patients. **(A)** PLS-DA was done out based only on the lipoprotein data panel (B.I. LISA™). The 4-group distribution was shown using the coordinates of loading components 1 and 2. **(B)** The values that contributed the most to these VIP scores are shown here by the subplot. **(C)** Lipoprotein data variables’ average trends were presented by sub-plot. **(D)** oPLS-DA study was performed, and LTCS vs control patients (HC) were compared. This analysis was done out based only on the lipoprotein data panel (B.I. LISA™). The two-group distribution was shown using the coordinates of loading components 1 and 2. **(E, F)** The values that contributed the most to these VIP scores and S-plot data of the regression model are shown here by the subplots, which are sorted from most significant to least significant. **(G)** Lastly, the lipoprotein panel Volcano analysis results showing trend-like (FC > 1.2, p (FDR-adjusted) ≤ 0.10) changes in ratio of LTCS/HC as presented by sub-plot. **(H)** Lastly, the main lipoprotein panel variables’ average trends were presented by sub-plot **(H)**. For each patient group (Recov (EDTA plasma) n=12, HC (serum or heparin plasma) n=73, LTCS (EDTA plasma) n=33, Acute (Heparin plasma) n=16 samples), an average normalized (scaled 0 to 1, averages were divided by a maximal average per variable).

Several studies on mild/moderate and acute COVID-19 patients have implicated the importance of lipoproteins in disease development (12, 30, 34, 37, 48–50). In our study, the four cohort groups based on lipoprotein parameters were partially separated by the PCA (**Figure 3A**, PCA loadings plot – **Supplementary Figure 4**). The Recov group was characterized by the highest levels of LDL-5 and LDL-6 subfraction cholesterol content (**Figure 3B**). By contrast, lipoproteins such as, V5FC, V5CH, and L6TG were increased mostly in LTCS patients (**Figures 3B, C**). We also identified that several lipoproteins were present in lower amounts in LTCS compared with Recov patients (**Supplementary Figure 5**). Herein, we found that HDL-4 triglycerides were considerably higher in the Recov group compared with the LTCS group.

Due to the small n-number in the Recov group we focused only on HC and LTCS patients. Here we observed in the oPLS-DA plots that HC and LTCS form two clusters though not being entirely separated (**Figure 3D**). Variable projection regression analysis revealed that a greater number of lipoproteins were highly abundant in HC compared to LTCS patients (**Figures 3E, F**). Based on volcano plots, we identified that 18 lipoproteins were increased whilst 34 lipoproteins were decreased in LTCS patients compared with HC (**Figure 3G**). On the Volcano analysis plot, we observed decrease in Apo-A2 (TPA2) and (fold changes > 1.2, p values (FDR-adjusted) ≤0.10) increased triglycerides. We carefully observed the substantial differences in several metabolites between LTCS and severe acute COVID-19 patients, including HDL cholesterol and apolipoprotein B100 Apo-B (TPAB) (**Figure 3H**).

Performing an additional analysis based on the Wilcoxon Rank Test (**Supplementary Table 4**, FDR-adjusted significances shown), we identified that Recov (*), acute (****), and LTCS (****) patients had higher blood triglycerides than the HC group, something that has been reported for COVID-positive individuals previously (12, 32, 48). Moreover, no lipoproteins (according to the FDR p values), while very-low-density lipoprotein (VLDL) phospholipids were elevated in LTCS (***, **Supplementary Table 5**, FDR-adjusted significances shown). Interestingly, free cholesterol levels were not significantly different between LTCS and acute COVID-19 groups. Of note, the acute COVID-19 group showed the highest blood triglyceride levels versus the Recov (**) and LTCS (**) groups (**Supplementary Table 4**).

### The combination of metabolites, lipoproteins and cytokines orchestrates pathological phenotypes

Several studies reported that inflammation, metabolism, and lipoprotein parameters act in unison to overall inform about the specific disease status such as mild, moderate, or severe. This knowledge thus can be used to predict and stratify disease severity (2, 3, 21, 37, 49, 51–54). Indeed, our cytokine and chemokine profiling showed that acute COVID-19 samples showed a trend of highest levels of cytokines & chemokines compared to either HC, LTCS or Recov (**Supplementary Table 6**). Furthermore, most of the cytokines and chemokines had a tendency of lower levels in either the LTCS or Recov group compared with HC, except IL-18 chemokine. In fact, IL-18 (mean value, compared *via* the Tukey’s multiple comparisons test computing adjusted p values) was found to be higher in LTCS and Recov compared with HC, however not reaching a significance level (**Supplementary Figure 6**; **Supplementary Tables 6**, **7**). We validated previously published data that IL-8 chemokine and IL-6 and IL-10 cytokines were abundantly present in acute COVID-19 patients (55, 56). With our data we also performed PCA and PLS-DA analysis and could identify a major separation among acute and LTCS patients (**Supplementary Figure 7A**).

Notably, in a correlation analysis of cytokines, chemokines and metabolites revealed that acute COVID-19 patients showed the highest levels of cytokines (**Supplementary Figure 7B**). The Recov patients showed medium levels compared with LTCS, whereas all these cytokines and chemokines were present in low abundance in LTCS patients in overall comparison (**Supplementary Figures 7B, C**). A key observation is high citrate, histidine and ornithine abundance in LTCS patients compared with any other group (HC, Recov, and acute) (**Supplementary Figures 7B, C** and **Supplementary Table 8**). Furthermore, Spearman correlation analysis was performed (**Supplementary Figure 7D**, shown correlations are FDR-corrected p (FDR) < 0.005) to identify possible interactions among cytokines, chemokines, metabolites and lipoproteins (**Supplementary Table 9**). In doing so, we identified relatively high negative correlations against 2-aminobutyrate (2-AB), an antioxidant synthesis controlling metabolite (57), alanine, threonine, pyruvate, tyrosine, sarcosine, ornithine, glutamine, citrate, and several additional cytokine panel parameters (IL-10/23/12p70/8/33/6/1b/18/17A, INF-g, IFN-a2, TNF-a, and MCP-1). In the other hand, the amino acid histidine was also highly elevated in the LTCS group. These results indicate a metabolic shift in LTCS individuals. Some of these findings above were confirmed in previously published studies (29, 58). Our interpretation is that with deterioration in health, phenylalanine and histidine concentrations increased, as did ketone body levels (48). We believe that these results are novel regarding LTCS patients.

**Figure 4:**
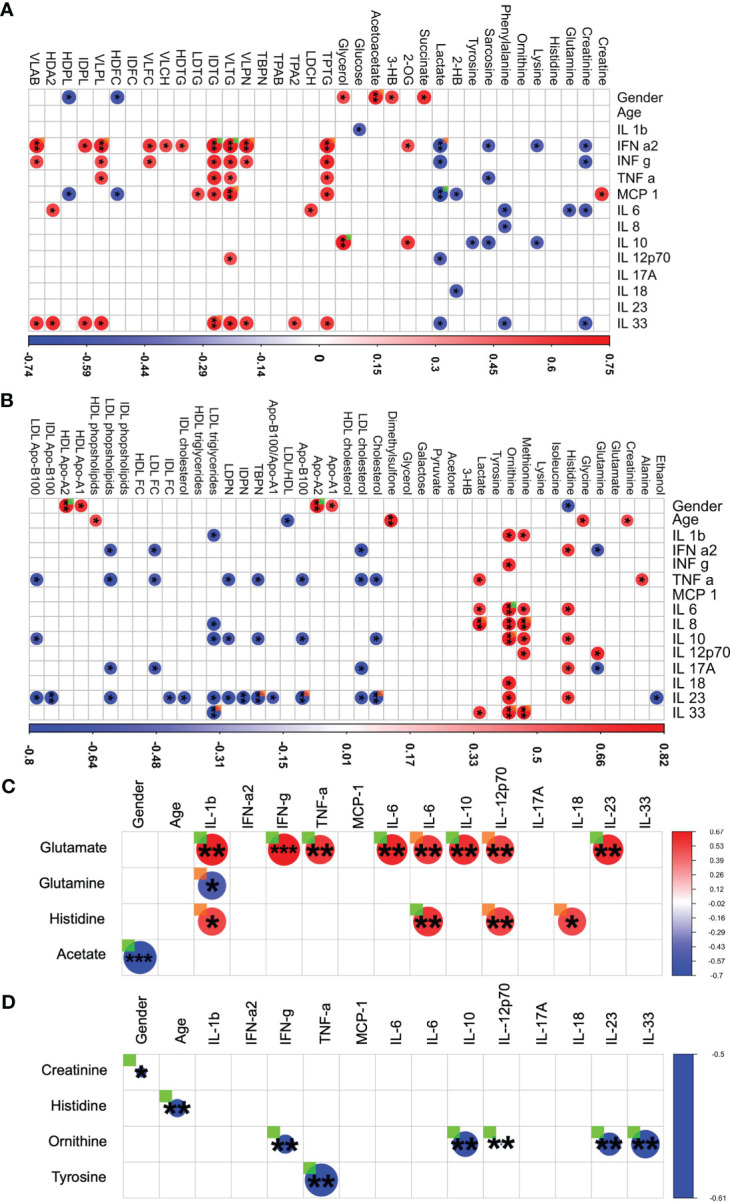
Integrated analysis of metabolites, lipoproteins, chemokines and cytokines in LTCS and comparators groups. For each patient group (Recov (EDTA plasma) n=11, HC (Heparin plasma) n=32, LTCS (EDTA plasma) n=24, acute (Heparin plasma) n=15 samples), based on the cytokine data availability, Spearman correlation test with exact p values (r values of the correlational analysis scaled -1 to 1, colored blue to red respectively) was conducted. Based on measurable metabolites data (B.I. QUANT-PS™) and a selection of lipoprotein parameter list data (B.I. LISA™). **(A)** The graphical representation is performed with filters of |r| ≥ 0.500 and p < 0.05 has been shown in panels – acute group, **(B)** – Recov group, **(C)** – LTCS group, **(D)** – HC group). Correlational values for Ca-EDTA and K-EDTA not shown. ▪ (ORANGE) – FDR-adjected p value of a correlation is < 0.10 in a patient group; ▪ (GREEN) – Significant, FDR-adjected p value of a correlation is < 0.05 in a patient group. A statistically significant difference was indicated when the value of raw P was less than 0.05 (* - P ≤ 0.05, ** - P ≤ 0.01, *** - P ≤ 0.001).

One further interesting finding was based on investigating the impact of gender on LCTS. Spearman correlation analysis with thresholds of |r| ≥ 0.5 and p < 0.05 demonstrates (**Figure 4**) that the acute group appeared to have a strong gender-based bias positive to succinate, glycerol, 3-hydroxybutyrate (3-HB), acetoacetate; and a negative correlation towards HDL free cholesterol, lactate, and phospholipids (**Figure 4A**). Several cytokine panel data entries had a positive correlation especially with VLDL triglycerides (**Figure 4A**). A negative correlation among cytokines and creatinine together with sarcosine was also observed. Also, the macrophage attractant chemokine protein MCP-1 had a special correlational profile dedicated in a r positive towards creatine and VLDL triglycerides whilst, r negative for the correlations with lactate, HDL free cholesterol and phospholipids. These findings highlight a complex nexus in acute COVID-19 patients among inflammation and metabolic regulation.

We were further interested to decipher and understand a correlation for the Recov and LTCS patients. We were able to identify a strong positive correlation among glutamate (with TNF-a), ornithine, lactate (with IL-8), and pyruvate (with IFN-a2) (**Figure 4B**). In contrast, lipoproteins showed a mostly negative correlation to cytokines. A gender-based bias positive correlation was identified for apolipoproteins A1 and A2 whilst, histidine negatively correlated with gender. Age appeared to be positively associated with HDL cholesterol and negatively with overall blood LDL/HDL lipoproteins fraction ratio (**Figure 4B**).

In case of LTCS patients some unique findings were identified. We were able to determine that a large set of cytokines were changing in a similar way to Recov amongst patients as histidine and glutamate (**Figure 4C**). Negative associations were found for glutamine and IL-1b. A strong gender/age-based bias was found for acetate in LTCS patients.

**Figure 5:**
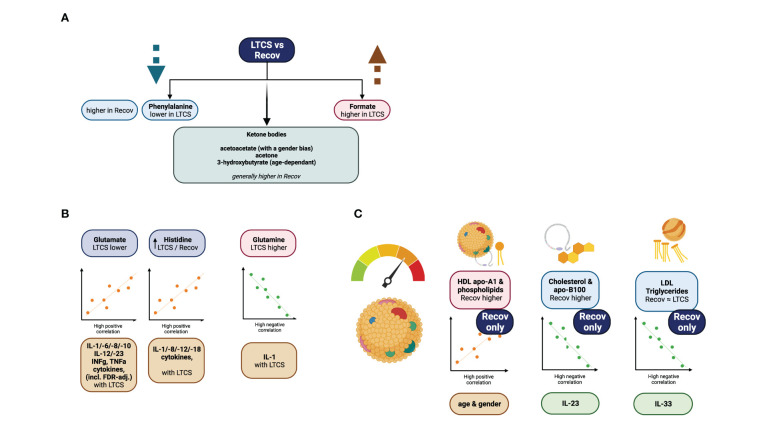
Graphical summary of the study. **(A)** focuses on phenylalanine, and formate as significant variables identified *via* regression model analysis. **(B)** demonstrates further strong metabolite correlations with the cytokine data. **(C)** is showing highlights of the lipoprotein data analysis.

In case of HC only two major negative correlations were found for the healthy controls: creatinine – gender and ornithine – IL-12p70 (**Figure 4D**). An overall graphical summary of our key findings is provided in **Figure 5** and **Supplementary Table 10**. Thus, it seems that each disease state has its own bubble network to combat the virus and regulate the function of host system.

## Discussion

LTCS is a condition which is thought to debilitate a person’s life after a SARS-CoV-2 viral infection and post-recovery for several months up to years. It is estimated that approximately 10-20% of all COVID-19 patients are susceptible to develop LTCS. Through our integrative approach of quantitative measurement of metabolites, lipoproteins, inflammation parameters and cytokines, we identified several features, which are uniquely dysregulated in LTCS patients (**Figure 5**). Our major findings revealed that lactate and pyruvate were highly upregulated in LTCS patients compared with HC and similar metabolites were also upregulated in Recov patients. This could be due to dysregulated oxidative phosphorylation in Recov or LTCS patients. Furthermore, phenylalanine, glycine, acetate, Gln/Glu ratio, glutamine, and creatinine were decreased in LTCS patients compared with HC or Recov. This may be indicative of the LTCS symptoms. A sign of the greater long COVID-related severity state could be further associated with phenylalanine, ketone bodies (acetoacetate, acetone, and 3-HB), formate, histidine, and glutamate blood levels (**Figure 5**). As there is a demand for the amino acid and its further pathway products, phenylalanine levels decay in the COVID recovery phase as reported previously (59), similarly to currently investigated LTCS group versus Recov comparison. In here, a slight change of acetoacetate could be an indicator of starvation-like conditions (60). This together with other statistically significant parameters could predict COVID-19 disease severity (3). From the correlational analysis, we were able to determine that correlations of glutamate, glutamine, and histidine were stronger to the cytokine data in the LTCS group. The findings therefore suggest a contrastingly higher role of glutamate to IL-1b, INF-g (predominantly), TNF-a, IL-8/10/18 cytokines positive correlations among the LTCS individuals. This finding is similar to the previously reported association of mild/acute COVID-19 patients metabolomic analysis and its classification to the cytokine panel data (10).

Whilst considering previously reported metabolic shifts for COVID-19-positive patients (15, 32, 59), these LTCS-specific metabolites - creatine and 3-HB can provide an insight which metabolic shifts could be persisting and represent a continuous risk to the patients’ health. Interestingly, the LTCS profiles of the metabolites citrate, 3-HB and histidine were changed in contrast to acute COVID-19 patient. Furthermore, LTCS patients had higher levels of formate and acetone than Recov patients. Since lactate and pyruvate levels in LTCS patients were significantly different from those of either HC or acute COVID-19 patients (to a lesser extent). We concluded that blood metabolic changes were primarily responsible for increased energy demand for the immune response, which was also prominent in individuals with severe respiratory distress (61). We detected a higher blood lactate level in LTCS patients, which may be associated with acidosis, which might explain symptoms such as fatigue and brain fog (62). Additionally, decreased blood glutamine levels that had previously been identified are crucial in acute and post-acute COVID and were advised to be supplemented to patients (33, 63). Our study is in line with the published studies and suggested that glutamine deficiency in the LTCS condition. Our finding could potentially helpful for evaluation of patients’ wellbeing (64).

Somehow as expected, Recov and LTCS patients showed very similar types of metabolic dysregulations. However, we identified some difference between the groups especially for formate, acetate, creatinine, due to the small n-number no significance level was achieved. Moreover, HDL-4 triglycerides and HDL-4 ApoA1 were considerably greater in the Recov group than in the LTCS group. While the smaller HDLs and their Apo-A1 content, e.g. HDL-4 were previously reported to indicate the severity of the pulmonary arterial hypertension condition (65), other research - in regards to the increased risk of COVID infection to arterial hypertension condition (66). Consequently, these lipoprotein modifications might be mapped and continue to reveal subtly altered triglycerides characteristics that depict COVID-associated dysregulation (48). When blood ketone body levels rise, so do triglyceride levels, which is more indication of an acute energy reliance.

We further identified an elevation of citrate and pyruvate in blood of the LTCS patient group compared with HC. This is in line with another study which identified higher levels of pyruvic acid accumulated in the blood stream of COVID-19 patients and which could be used to prognose disease severity (67). Further, greater levels of lactate in COVID-19 patients are an already established finding (68). We therefore hypothesize that glycolysis/gluconeogenesis and Krebs cycle metabolic pathways will lead to an elevated consumption of glucose to produce citric acid. It is interesting to note that citrate levels did not significantly correlate to any of the chemokine or cytokines, yet it was only connected to the gender factor. Therefore, gender based metabolic dysregulation could play an important role to understand the disease severity. This is especially important as certain LTCS symptoms have been reported more or even only for female or male patients.

Maintained triglyceride and other lipoproteins change indicate that COVID-19 like features persist in LTCS patients when comparisons were made with HC. Therefore, elevated apolipoproteins ratio B100 to A1 and overall blood triglycerides could be attributed to the disease group (12). Our data implies that HDL cholesterol (HDCH) is lowered in the LTCS patients. Previously, it was identified that severe immunosuppression is a key for the severity of COVID-19 rather than the cytokine storm (69). Thus, it is plausible that lower level of lipids and inflammatory cytokines may be of important for further disease symptoms in LTCS patients. Another correlation of the LTCS group to acute COVID-19 patients is noticed *via* lowered apolipoproteins A1 & A2 levels, among other close structures they had been lowered in ill subjects (70, 71).

## Conclusion and limitation of the study

In this study we compared quantitative NMR serum parameters of four different groups. One obvious limitation was the low number of samples in the “acute” (n = 16) and “recovered” (n = 12) group. The focus was thus set on the discussion of results obtained from the comparison between healthy controls (n = 73) and LTCS (n = 33). These numbers are suitable for a statistical metabolomics approach based on IVDr-NMR spectroscopy. We identified a large set of quantitative NMR data on metabolites and lipoproteins and inflammation parameters in LTCS patients and highlight that glutamate, citrate, lactate, pyruvate, histidine, HDL (HDL-4) and total blood triglycerides, HDL (HDL-4) apolipoproteins Apo-A1, IL-18, TNF-a, IL-23, IL-8, MCP-1 could be key parameters in the pathophysiology of maintained disease symptoms and even progression. It should be noted as a limitation, that for this analysis only a very basic set of patient metadata was available. Thus, the estimation of the role of underlying comorbidities and the comparability to healthy controls is limited. Especially with severe COVID-19 patients (i.e. those who were hospitalized) it can be assumed that a majority of them had risk factors like diabetes, obesity, hypertension, etc. – adjustment for these risk factors in the “healthy controls” would be very interesting. The core of this study therefore was based on the two larger groups LTCS and HC, however also within LTCS comorbidities might have contributed to changes. Furthermore, the limited number and only single time point of samples for the Recov and acute group should be considered when comparing the results of this study with similar projects. Another clear limitation of the study is the age difference between Recov and all other groups, as it is known that age-related metabolic disorders such as diabetes or hypertension have an impact on the blood metabolome and lipoproteome. Furthermore we want to point out the difference in the delays between the acute COVID-19 infection diagnosis and the plasma collection in between Recov and LTCS group as another potential confounder. Nonetheless, our results confirm and align with some of the previously published results and show novel insights into persisting altered blood metabolome, lipoproteome and inflammation parameters when comparing healthy controls with LTCS specimen.

## Data availability statement

The original contributions presented in the study are included in the article/**Supplementary Material**. Further inquiries can be directed to the corresponding authors.

## Ethics statement

The studies involving human participants were reviewed and approved by COVID-19 NGS; Ethics number: 286/2020B1 and Clinical Trial number: NCT04364828. According the declaration of Helsinki, the patients/participants provided their written informed consent to participate in this study.

## Author contributions

GB: Performed the NMR spectroscopy experiments, processed the samples, analyzed the integrated data, figure preparation, manuscript writing. RB: Performed the experiments, patient recruitment and processing of blood plasma. AL, SG, HH, KK, MB: Patient recruitment, blood sample collection, patient metadata collection, editing the manuscript. CC, HS: provided control samples and IVDr software license. CT: Planned the experiments, performed the NMR spectroscopy experiments, processed the samples, analyzed the integrated data, figure preparation, manuscript editing. YS: Overall project management and execution, planned the experiments, cytokine assay and data analysis, preparation of the final figures, manuscript writing. All authors contributed to the article and approved the submitted version.

## Supplementary material

Supplementary Figure 1 The loadings plot of the principal component analysis (PCA) of the metabolomics data that were examined using PCA for the full cohort is shown in **Figure 2B**.

Supplementary Figure 2 Identification of metabolites in LTCS and Recov patients *via* o PLS-DA studies. This analysis was performed based only on quantifiable metabolites data (B.I. QUANT-PS™). The 4-group distribution was shown in **(A, B)** using the coordinates of T score and orthogonal T score. The values that contributed the most to these VIP scores and S plot loadings of the regression model are shown here by the subplot **(C)**. Lastly, the metabolite panel variables’ average trends were presented by sub-plot **(D)**.

Supplementary Figure 3 Pathway analysis of dysregulated metabolites and lipoproteins. For each patient group pairs (Recov (EDTA plasma) n=12, HC (Heparin plasma) n=32, LTCS (EDTA plasma) n=33, acute (Heparin plasma) n=16 samples), a number of metabolic pathways, as shown on the subplots **(A–F)**, related to the measurable metabolites data (B.I. QUANT-PS™) were identified in the patient groups. Predicted metabolic pathways are listed with p-values after the FDR applied (false discovery rate correction). The group distribution whose statistical importance has been shown in panels. Statistical significances: ns p > 0.05, * p ≤ 0.05, ** p ≤ 0.01, *** p ≤ 0.001, **** p ≤ 0.0001. ▪ (RED) - higher average levels in a patient group; ▪ (CYAN) – lower average levels in a patient group. TCA cycle – tricarboxylic acid cycle. CoA – coenzyme A. ATP – adenosine triphosphate. ADP – adenosine diphosphate. SCFA – short-chain fatty acids.

Supplementary Figure 4 Analysis supplement based on the PLS-DA. As illustrated in **Figure 3A, B**, the sub-plot is a depiction of the applied lipoprotein parameters in the PLS-DA analysis loadings coordinates for components 1 and 2.

Supplementary Figure 5 oPLS-DA study was performed, and LTCS vs recovered patients (Recov) were compared. This analysis was performed based only on lipoprotein data panel (B.I. LISA™). The two-group distribution was shown in **(A)** using the coordinates of loading components 1 and 2. The values that contributed the most to these VIP scores and S-plot data of the regression model are shown here by the subplots **(B)** and **(C)**, which are sorted from most significant to least significant. Lastly, the metabolite panel regression model analysis highlighted results (VIP > 1.0) visualized *via* a Heat map as presented by sub-plot **(D)**.

Supplementary Figure 6 IL-18 concentration levels of the subjects. Statistical significances: ns p > 0.05, * p ≤ 0.05, ** p ≤ 0.01.

Supplementary Figure 7 PLS-DA study was performed, and the available data from the cytokine panel table (**Supplementary Table 6**) were compared. The group distribution was shown in **(A)**. The values that contributed the most to these loadings of the regression model are shown here by the subplots **(B)** by Component 1) and **(C)** by Component 2, which are sorted from most significant to least significant *via* variables in projection score (VIP) plots. Lastly, variables’ panel of Spearman correlations for IL-10 cytokines showed trends against the main cytokine parameters by sub-plot **(D)**.

## References

[1] WHO Live Dashboard. (2020). Available at: https://covid19.who.int.

[2] Ceballos FC Virseda-Berdices A Resino S Ryan P Martinez-Gonzalez O Perez-Garcia F. Metabolic profiling at COVID-19 onset shows disease severity and sex-specific dysregulation. Front Immunol (2022) 13:925558. doi: 10.3389/fimmu.2022.925558 35844615 PMC9280146

[3] Lee JW Su Y Baloni P Chen D Pavlovitch-Bedzyk AJ Yuan D. Integrated analysis of plasma and single immune cells uncovers metabolic changes in individuals with COVID-19. Nat Biotechnol (2022) 40:110–20. doi: 10.1038/s41587-021-01020-4 PMC9206886 34489601

[4] Singh Y Trautwein C Fendel R Krickeberg N Berezhnoy G Bissinger R. SARS-CoV-2 infection paralyzes cytotoxic and metabolic functions of the immune cells. Heliyon (2021) 7:e07147. doi: 10.1016/j.heliyon.2021.e07147 34075347 PMC8159709

[5] Liu X Zhao J Wang H Wang W Su X Liao X. Metabolic defects of peripheral T cells in COVID-19 patients. J Immunol (2021) 206:2900–8. doi: 10.4049/jimmunol.2100068 34049969

[6] O’Carroll SM O’Neill LAJ. Targeting immunometabolism to treat COVID-19. Immunother Adv (2021) 1:ltab013. doi: 10.1093/immadv/ltab013 34240083 PMC8195165

[7] Cornillet M Strunz B Rooyackers O Ponzetta A Chen P Muvva JR. COVID-19-specific metabolic imprint yields insights into multiorgan system perturbations. Eur J Immunol (2022) 52:503–10. doi: 10.1002/eji.202149626 PMC9015354 34837225

[8] Jia H Liu C Li D Huang Q Liu D Zhang Y. Metabolomic analyses reveal new stage-specific features of COVID-19. Eur Respir J (2022) 59(2):2100284. doi: 10.1183/13993003.00284-2021 34289974 PMC8311281

[9] Costanzo M Caterino M Fedele R Cevenini A Pontillo M Barra L. COVIDomics: the proteomic and metabolomic signatures of COVID-19. Int J Mol Sci (2022) 23(5):2414. doi: 10.3390/ijms23052414 35269564 PMC8910221

[10] Danlos FX Grajeda-Iglesias C Durand S Sauvat A Roumier M Cantin D. Metabolomic analyses of COVID-19 patients unravel stage-dependent and prognostic biomarkers. Cell Death Dis (2021) 12:258. doi: 10.1038/s41419-021-03540-y 33707411 PMC7948172

[11] Wu D Shu T Yang X Song J-X Zhang M Yao C. Plasma metabolomic and lipidomic alterations associated with COVID-19. Natl Sci Rev (2020) 7(7):1157–68. doi: 10.1093/nsr/nwaa086 PMC7197563 34676128

[12] Lodge S Nitschke P Kimhofer T Coudert JD Begum S Bong SH. NMR spectroscopic windows on the systemic effects of SARS-CoV-2 infection on plasma lipoproteins and metabolites in relation to circulating cytokines. J Proteome Res (2021) 20:1382–96. doi: 10.1021/acs.jproteome.0c00876 33426894

[13] Wan Q Chen M Zhang Z Yuan Y Wang H Hao Y. Machine learning of serum metabolic patterns encodes asymptomatic SARS-CoV-2 infection. Front Chem (2021) 9:746134. doi: 10.3389/fchem.2021.746134 34660538 PMC8517325

[14] Hassan MA Al-Sakkaf K Shait Mohammed MR Dallol A Al-Maghrabi J Aldahlawi A. Integration of transcriptome and metabolome provides unique insights to pathways associated with obese breast cancer patients. Front Oncol (2020) 10:804. doi: 10.3389/fonc.2020.00804 32509585 PMC7248369

[15] Su Y Chen D Yuan D Lausted C Choi J Dai CL. Multi-omics resolves a sharp disease-state shift between mild and moderate COVID-19. Cell (2020) 183:1479–1495 e20. doi: 10.1016/j.cell.2020.10.037 33171100 PMC7598382

[16] He F Zhang T Xue K Fang Z Jiang G Huang S. Fecal multi-omics analysis reveals diverse molecular alterations of gut ecosystem in COVID-19 patients. Anal Chim Acta (2021) 1180:338881. doi: 10.1016/j.aca.2021.338881 34538334 PMC8310733

[17] Lv L Jiang H Chen Y Gu S Xia J Zhang H. The faecal metabolome in COVID-19 patients is altered and associated with clinical features and gut microbes. Anal Chim Acta (2021) 1152:338267. doi: 10.1016/j.aca.2021.338267 33648648 PMC7847702

[18] Wang C Li X Ning W Gong S Yang F Fang C. Multi-omic profiling of plasma reveals molecular alterations in children with COVID-19. Theranostics (2021) 11:8008–26. doi: 10.7150/thno.61832 PMC8315065 34335977

[19] Wu P Chen D Ding W Wu P Hou H Bai Y. The trans-omics landscape of COVID-19. Nat Commun (2021) 12:4543. doi: 10.1038/s41467-021-24482-1 34315889 PMC8316550

[20] Song Z Bao L Deng W Liu J Ren E Lv Q. Integrated histopathological, lipidomic, and metabolomic profiles reveal mink is a useful animal model to mimic the pathogenicity of severe COVID-19 patients. Signal Transduct Target Ther (2022) 7:29. doi: 10.1038/s41392-022-00891-6 35091528 PMC8795751

[21] Chen YM Zheng Y Yu Y Wang Y Huang Q Qian F. Blood molecular markers associated with COVID-19 immunopathology and multi-organ damage. EMBO J (2020) 39:e105896. doi: 10.15252/embj.2020105896 33140861 PMC7737620

[22] Bizkarguenaga M Bruzzone C Gil-Redondo R SanJuan I Martin-Ruiz I Barriales D. Uneven metabolic and lipidomic profiles in recovered COVID-19 patients as investigated by plasma NMR metabolomics. NMR BioMed (2022) 35:e4637. doi: 10.1002/nbm.4637 34708437 PMC8646702

[23] Falck-Jones S Vangeti S Yu M Falck-Jones R Cagigi A Badolati I. Functional monocytic myeloid-derived suppressor cells increase in blood but not airways and predict COVID-19 severity. J Clin Invest (2021) 131(6):e144734. doi: 10.1172/JCI144734 33492309 PMC7954608

[24] Abers MS Delmonte OM Ricotta EE Fintzi J Fink DL de Jesus AAA. An immune-based biomarker signature is associated with mortality in COVID-19 patients. JCI Insight (2021) 6(1):e144455. doi: 10.1172/jci.insight.144455 33232303 PMC7821609

[25] Al-Aly Z Bowe B Xie Y. Long COVID after breakthrough SARS-CoV-2 infection. Nat Med (2022) 28:1461–7. doi: 10.1038/s41591-022-01840-0 PMC9307472 35614233

[26] Guntur VP Nemkov T de Boer E Mohning MP Baraghoshi D Cendali FI. Signatures of mitochondrial dysfunction and impaired fatty acid metabolism in plasma of patients with post-acute sequelae of COVID-19 (PASC). Metabolites (2022) 12(11):1026. doi: 10.3390/metabo12111026 36355108 PMC9699059

[27] Correa HL Deus LA Araujo TB Reis AL Amorim CEN Gadelha AB. Phosphate and IL-10 concentration as predictors of long-covid in hemodialysis patients: a Brazilian study. Front Immunol (2022) 13:1006076. doi: 10.3389/fimmu.2022.1006076 36248863 PMC9562993

[28] Letertre MPM Giraudeau P de Tullio P. Nuclear magnetic resonance spectroscopy in clinical metabolomics and personalized medicine: current challenges and perspectives. Front Mol Biosci (2021) 8:698337. doi: 10.3389/fmolb.2021.698337 34616770 PMC8488110

[29] Kimhofer T Lodge S Whiley L Gray N Loo RL Lawler NG. Integrative modeling of quantitative plasma lipoprotein, metabolic, and amino acid data reveals a multiorgan pathological signature of SARS-CoV-2 infection. J Proteome Res (2020) 19:4442–54. doi: 10.1021/acs.jproteome.0c00519 32806897

[30] Correia BSB Ferreira VG Piagge PMFD Almeida MB Assunção NA Raimundo JRS. 1H qNMR-based metabolomics discrimination of covid-19 severity. J Proteome Res (2022) 21:1640–53. doi: 10.1021/acs.jproteome.1c00977 35674498

[31] Ghini V Maggi L Mazzoni A Spinicci M Zammarchi L Bartoloni A. Serum NMR profiling reveals differential alterations in the lipoproteome induced by pfizer-BioNTech vaccine in COVID-19 recovered subjects and naïve subjects. Front Mol Biosci (2022) 9:839809. doi: 10.3389/fmolb.2022.839809 35480886 PMC9037139

[32] Meoni G Ghini V Maggi L Vignoli A Mazzoni A Salvati L. Metabolomic/lipidomic profiling of COVID-19 and individual response to tocilizumab. PloS Pathog (2021) 17:e1009243. doi: 10.1371/journal.ppat.1009243 33524041 PMC7877736

[33] Holmes E Wist J Masuda R Lodge S Nitschke P Kimhofer T. Incomplete systemic recovery and metabolic phenoreversion in post-Acute-Phase nonhospitalized COVID-19 patients: implications for assessment of post-acute COVID-19 syndrome. J Proteome Res (2021) 20:3315–29. doi: 10.1021/acs.jproteome.1c00224 34009992

[34] Masuda R Lodge S Nitschke P Spraul M Schaefer H Bong SH. Integrative modeling of plasma metabolic and lipoprotein biomarkers of SARS-CoV-2 infection in Spanish and Australian COVID-19 patient cohorts. J Proteome Res (2021) 20:4139–52. doi: 10.1021/acs.jproteome.1c00458 34251833

[35] Nitschke P Lodge S Hall D Schaefer H Spraul M Embade N. Direct low field J-edited diffusional proton NMR spectroscopic measurement of COVID-19 inflammatory biomarkers in human serum. Analyst (2022) 147:4213–21. doi: 10.1039/D2AN01097F 35994017

[36] Schmelter F Föh B Mallagaray A Rahmöller J Ehlers M Lehrian S. Metabolic and lipidomic markers differentiate COVID-19 from non-hospitalized and other intensive care patients. Front Mol Biosci (2021) 8:737039. doi: 10.3389/fmolb.2021.737039 34938772 PMC8686182

[37] Rossler T Berezhnoy G Singh Y Cannet C Reinsperger T Schafer H. Quantitative serum NMR spectroscopy stratifies COVID-19 patients and sheds light on interfaces of host metabolism and the immune response with cytokines and clinical parameters. Metabolites (2022) 12(12):1277. doi: 10.3390/metabo12121277 36557315 PMC9781847

[38] Pang Z Chong J Zhou G de Lima Morais DA Chang L Barrette M. MetaboAnalyst 5.0: narrowing the gap between raw spectra and functional insights. Nucleic Acids Res (2021) 49:W388–96. doi: 10.1093/nar/gkab382 PMC8265181 34019663

[39] Stacklies W Redestig H Scholz M Walther D Selbig J. pcaMethods–a bioconductor package providing PCA methods for incomplete data. Bioinformatics (2007) 23:1164–7. doi: 10.1093/bioinformatics/btm069 17344241

[40] Dieterle F Ross A Schlotterbeck G Senn H. Probabilistic quotient normalization as robust method to account for dilution of complex biological mixtures. Appl 1H NMR metabonom Anal Chem (2006) 78:4281–90. doi: 10.1021/ac051632c 16808434

[41] Pang Z Zhou G Chong J Xia J. Comprehensive meta-analysis of COVID-19 global metabolomics datasets. Metabolites (2021) 11(1):44. doi: 10.3390/metabo11010044 33435351 PMC7827862

[42] Venkatesan P. NICE guideline on long COVID. Lancet Respir Med (2021) 9(2):129. doi: 10.1016/S2213-2600(21)00031-X 33453162 PMC7832375

[43] Karu N Kindt A van Gammeren AJ Ermens AAM Harms AC Portengen L. Severe COVID-19 is characterised by perturbations in plasma amines correlated with immune response markers, and linked to inflammation and oxidative stress. Metabolites (2022) 12(7):618. doi: 10.3390/metabo12070618 35888742 PMC9321395

[44] Kaur G Ji X Rahman I. SARS-CoV2 infection alters tryptophan catabolism and phospholipid metabolism. Metabolites (2021) 11(10):659. doi: 10.3390/metabo11100659 34677374 PMC8538244

[45] Krishnan S Nordqvist H Ambikan AT Gupta S Sperk M Svensson-Akusjarvi S. Metabolic perturbation associated with COVID-19 disease severity and SARS-CoV-2 replication. Mol Cell Proteomics (2021) 20:100159. doi: 10.1016/j.mcpro.2021.100159 34619366 PMC8490130

[46] Lewis HM Liu Y Frampas CF Longman K Spick M Stewart A. Metabolomics markers of COVID-19 are dependent on collection wave. Metabolites (2022) 12(8):713. doi: 10.3390/metabo12080713 36005585 PMC9415837

[47] Dona AC Jiménez B Schäfer H Humpfer E Spraul M Lewis MR. Precision high-throughput proton NMR spectroscopy of human urine, serum, and plasma for Large-scale metabolic phenotyping. Anal Chem (2014) 86:9887–94. doi: 10.1021/ac5025039 25180432

[48] Bruzzone C Bizkarguenaga M Gil-Redondo R Diercks T Arana E Garcia de Vicuna A. SARS-CoV-2 infection dysregulates the metabolomic and lipidomic profiles of serum. iScience (2020) 23:101645. doi: 10.1016/j.isci.2020.101645 33043283 PMC7534591

[49] Gafson AR Thorne T McKechnie CIJ Jimenez B Nicholas R Matthews PM. Lipoprotein markers associated with disability from multiple sclerosis. Sci Rep (2018) 8:17026. doi: 10.1038/s41598-018-35232-7 30451923 PMC6242870

[50] Sindelar M Stancliffe E Schwaiger-Haber M Anbukumar DS Adkins-Travis K Goss CW. Longitudinal metabolomics of human plasma reveals prognostic markers of COVID-19 disease severity. Cell Rep Med (2021) 2:100369. doi: 10.1016/j.xcrm.2021.100369 34308390 PMC8292035

[51] Buyukozkan M Alvarez-Mulett S Racanelli AC Schmidt F Batra R Hoffman KL. Integrative metabolomic and proteomic signatures define clinical outcomes in severe COVID-19. iScience (2022) 25:104612. doi: 10.1016/j.isci.2022.104612 35756895 PMC9212983

[52] Ghini V Meoni G Pelagatti L Celli T Veneziani F Petrucci F. Profiling metabolites and lipoproteins in COMETA, an Italian cohort of COVID-19 patients. PloS Pathog (2022) 18:e1010443. doi: 10.1371/journal.ppat.1010443 35446921 PMC9022834

[53] Kovarik JJ Bileck A Hagn G Meier-Menches SM Frey T Kaempf A. Multi-omics provide evidence for an anti-inflammatory immune signature and metabolic alterations in patients with Long COVID Syndrome – an exploratory study. medRxiv, preprint (2022). doi: 10.1371/journal.ppat.1010443

[54] Scherer PE Kirwan JP Rosen CJ. Post-acute sequelae of COVID-19: a metabolic perspective. Elife (2022) 11:e78200. doi: 10.7554/eLife.78200 35318939 PMC8942467

[55] Liu J Li S Liu J Liang B Wang X Wang H. Longitudinal characteristics of lymphocyte responses and cytokine profiles in the peripheral blood of SARS-CoV-2 infected patients. EBioMedicine (2020) 55:102763. doi: 10.1016/j.ebiom.2020.102763 32361250 PMC7165294

[56] Del Valle DM Kim-Schulze S Huang H-H Beckmann ND Nirenberg S Wang B. An inflammatory cytokine signature predicts COVID-19 severity and survival. Nat Med (2020) 26(10):1636–43. doi: 10.1038/s41591-020-1051-9 PMC7869028 32839624

[57] Irino Y Toh R Nagao M Mori T Honjo T Shinohara M. 2-aminobutyric acid modulates glutathione homeostasis in the myocardium. Sci Rep (2016) 6:36749. doi: 10.1038/srep36749 27827456 PMC5101505

[58] Shi D Yan R Lv L Jiang H Lu Y Sheng J. The serum metabolome of COVID-19 patients is distinctive and predictive. Metabolism (2021) 118:154739. doi: 10.1016/j.metabol.2021.154739 33662365 PMC7920809

[59] Ansone L Briviba M Silamikelis I Terentjeva A Perkons I Birzniece L. Amino acid metabolism is significantly altered at the time of admission in hospital for severe COVID-19 patients: findings from longitudinal targeted metabolomics analysis. Microbiol Spectr (2021) 9:e00338–21. doi: 10.1128/spectrum.00338-21 PMC8653833 34878333

[60] Watanabe M Balena A Masi D Tozzi R Risi R Caputi A. Central obesity improvement and blood glucose reduction are associated with a stronger adaptive immune response following COVID-19 mRNA vaccine. Vaccines (Basel) (2022) 10:79. doi: 10.3390/vaccines10010079 35062740 PMC8780354

[61] Yan Y Chen J Liang Q Zheng H Ye Y Nan W. Metabolomics profile in acute respiratory distress syndrome by nuclear magnetic resonance spectroscopy in patients with community-acquired pneumonia. Respir Res (2022) 23:172. doi: 10.1186/s12931-022-02075-w 35761396 PMC9235271

[62] Bateman L Bested AC Bonilla HF Chheda BV Chu L Curtin JM. Myalgic Encephalomyelitis/Chronic fatigue syndrome: essentials of diagnosis and management. Mayo Clinic Proc (2021) 96:2861–78. doi: 10.1016/j.mayocp.2021.07.004 34454716

[63] Mohajeri M Horriatkhah E Mohajery R. The effect of glutamine supplementation on serum levels of some inflammatory factors, oxidative stress, and appetite in COVID-19 patients: a case–control study. Inflammopharmacology (2021) 29:1769–76. doi: 10.1007/s10787-021-00881-0 PMC8552429 34709541

[64] Baranovicova E Bobcakova A Vysehradsky R Dankova Z Halasova E Nosal V. The ability to normalise energy metabolism in advanced COVID-19 disease seems to be one of the key factors determining the disease progression–a metabolomic NMR study on blood plasma. Appl Sci (2021) 11:4231. doi: 10.3390/app11094231

[65] Harbaum L Ghataorhe P Wharton J Jiménez B Howard LSG Gibbs JSR. Reduced plasma levels of small HDL particles transporting fibrinolytic proteins in pulmonary arterial hypertension. Thorax (2019) 74:380–9. doi: 10.1136/thoraxjnl-2018-212144 PMC6475111 30478197

[66] Kazenwadel J Berezhnoy G Cannet C Schäfer H Geisler T Rohlfing A-K. Stratification of hypertensive COVID-19 patients by quantitative NMR spectroscopy of serum metabolites, lipoproteins, inflammation markers. medRxiv, preprint, (2022). doi: 10.1101/2022.12.20.22283729

[67] Ceperuelo-Mallafre V Reverte L Peraire J Madeira A Maymo-Masip E Lopez-Dupla M. Circulating pyruvate is a potent prognostic marker for critical COVID-19 outcomes. Front Immunol (2022) 13:912579. doi: 10.3389/fimmu.2022.912579 36189213 PMC9515795

[68] Carpenè G Onorato D Nocini R Fortunato G Rizk JG Henry BM. Blood lactate concentration in COVID-19: a systematic literature review. Clin Chem Lab Med (2022) 60:332–7. doi: 10.1515/cclm-2021-1115 34856090

[69] Remy KE Mazer M Striker DA Ellebedy AH Walton AH Unsinger J. Severe immunosuppression and not a cytokine storm characterizes COVID-19 infections. JCI Insight (2020) 5(17):e140329. doi: 10.1172/jci.insight.140329 32687484 PMC7526441

[70] Messner CB Demichev V Wendisch D Michalick L White M Freiwald A. Ultra-High-Throughput clinical proteomics reveals classifiers of COVID-19 infection. Cell Syst (2020) 11:11–24 e4. doi: 10.1016/j.cels.2020.05.012 32619549 PMC7264033

[71] Shen B Yi X Sun Y Bi X Du J Zhang C. Proteomic and metabolomic characterization of COVID-19 patient sera. Cell (2020) 182:59–72 e15. doi: 10.1016/j.cell.2020.05.032 32492406 PMC7254001

