# Supplementary material for: Maintained imbalance of triglycerides, apolipoproteins, energy metabolites and cytokines in long-term COVID-19 syndrome patients
Source: Front Immunol. 2023 May 9;14:1144224. doi: 10.3389/fimmu.2023.1144224 (PMC10203989; doi:10.3389/fimmu.2023.1144224)
Supplement: Supplementary Figure 1 — The loadings plot of the principal component analysis (PCA) of the metabolomics data that were examined using PCA for the full cohort is shown in Figure 2B. [file DataSheet_1.pdf]

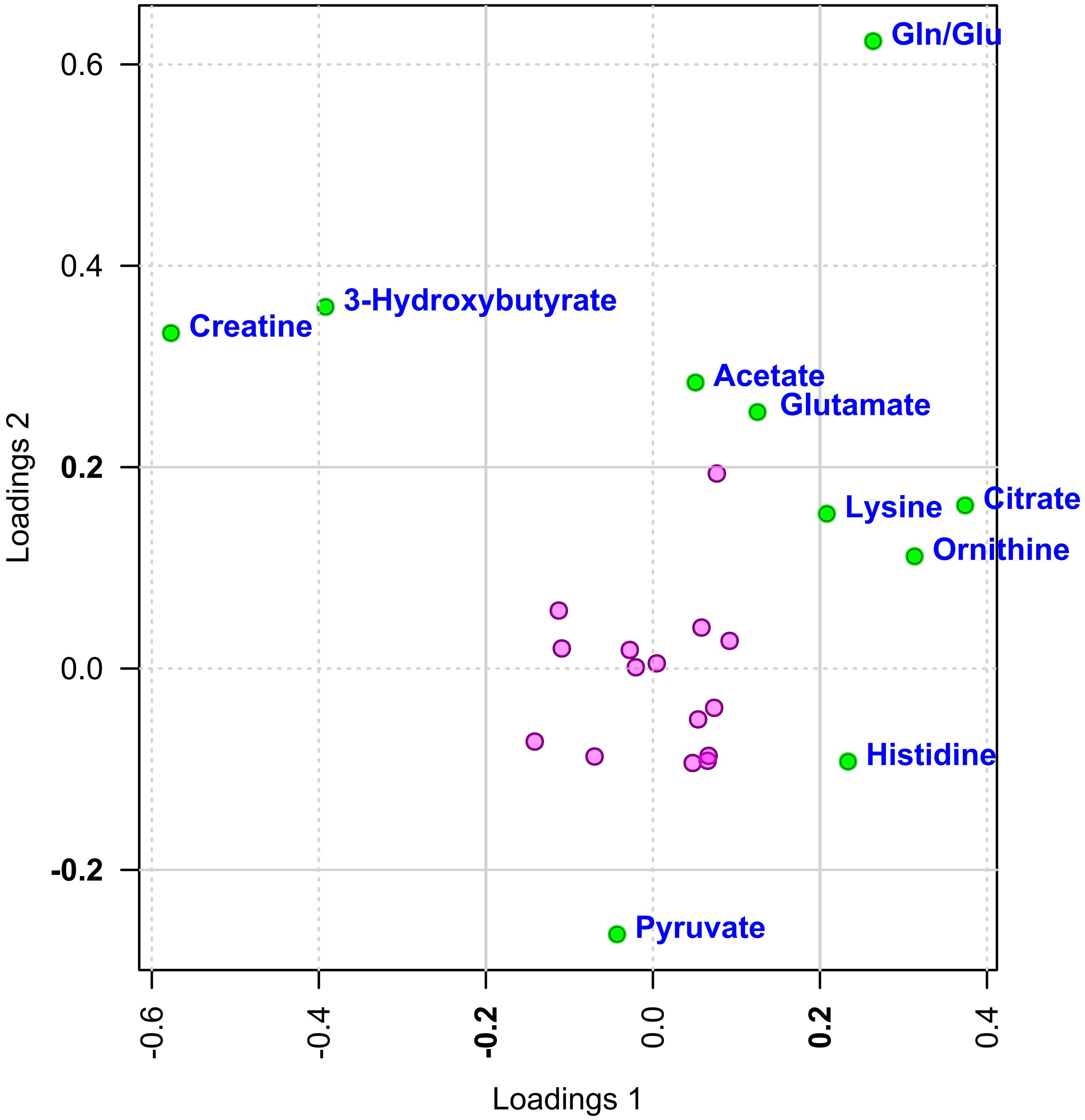

Gln/Glu
3-Hydroxybutyrate
Creatine
Acetate
Glutamate
Lysine
Citrate
Ornithine
Histidine
Pyruvate
Loadings 2
Loadings 1
0.6
0.4
0.2
0.0
-0.2
-0.6
-0.4
-0.2
0.0
0.2
0.4

Suppl. Fig. 1

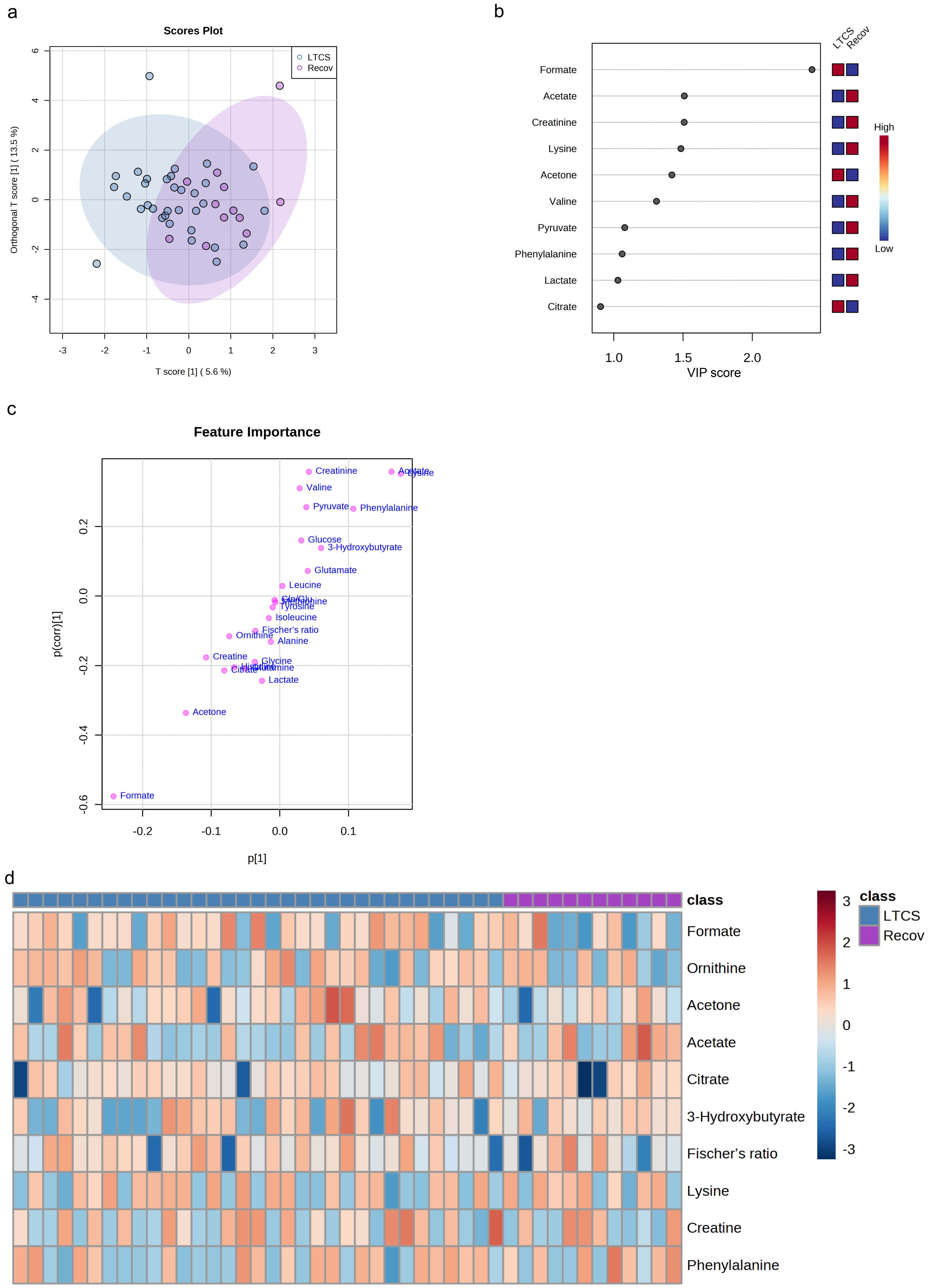
a
Scores Plot
LTCS
Recov
Orthogonal T score [1] (13.5 %)
T score [1] (5.6 %)
b
LTCS
Recov
Formate
Acetate
Creatinine
Lysine
Acetone
Valine
Pyruvate
Phenylalanine
Lactate
Citrate
High
Low
1.0
1.5
2.0
VIP score
c
Feature Importance
Creatinine
Acetate
Lysine
Valine
Pyruvate
Phenylalanine
Glucose
3-Hydroxybutyrate
Glutamate
Leucine
Methionine
Tyrosine
Isoleucine
Fischer's ratio
Ornithine
Alanine
Creatine
Glycine
Citrate
Lactate
Acetone
Formate
p(corr)[1]
p[1]
d
class
Formate
Ornithine
Acetone
Acetate
Citrate
3-Hydroxybutyrate
Fischer's ratio
Lysine
Creatine
Phenylalanine
class
LTCS
Recov

Suppl. Fig. 2

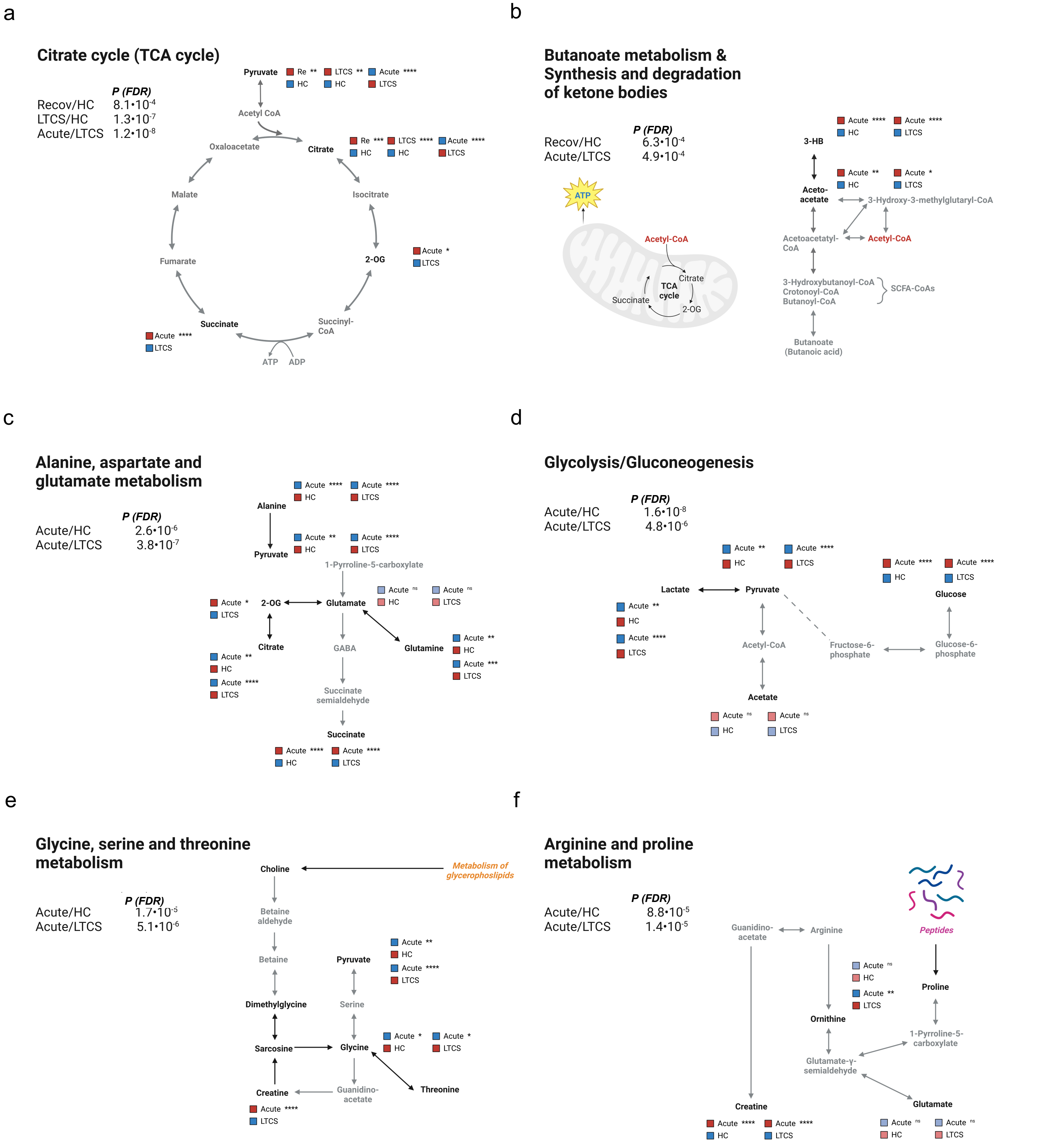
a
Citrate cycle (TCA cycle)
P (FDR)
Recov/HC $8.1 \cdot 10^{-4}$
LTCS/HC $1.3 \cdot 10^{-7}$
Acute/LTCS $1.2 \cdot 10^{-8}$
Pyruvate
Re ** HC
LTCS ** HC
Acute **** LTCS
Acetyl CoA
Oxaloacetate
Citrate
Re *** HC
LTCS **** HC
Acute **** LTCS
Isocitrate
Malate
2-OG
Acute * LTCS
Fumarate
Succinate
Acute **** LTCS
Succinyl-CoA
ATP
ADP
b
Butanoate metabolism & Synthesis and degradation of ketone bodies
P (FDR)
Recov/HC $6.3 \cdot 10^{-4}$
Acute/LTCS $4.9 \cdot 10^{-4}$
ATP
Acetyl-CoA
Citrate
TCA cycle
Succinate
2-OG
3-HB
Acute **** HC
Acute **** LTCS
Aceto-acetate
Acute ** HC
Acute * LTCS
3-Hydroxy-3-methylglutaryl-CoA
Acetoacetatyl-CoA
Acetyl-CoA
3-Hydroxybutanoyl-CoA
Crotonoyl-CoA
Butanoyl-CoA
SCFA-CoAs
Butanoate (Butanoic acid)
c
Alanine, aspartate and glutamate metabolism
P (FDR)
Acute/HC $2.6 \cdot 10^{-6}$
Acute/LTCS $3.8 \cdot 10^{-7}$
Alanine
Acute **** HC
Acute **** LTCS
Pyruvate
Acute ** HC
Acute **** LTCS
1-Pyrroline-5-carboxylate
Acute ns HC
Acute ns LTCS
Acute * LTCS
2-OG
Glutamate
Citrate
Acute ** HC
Acute **** LTCS
GABA
Glutamine
Acute ** HC
Acute *** LTCS
Succinate semialdehyde
Succinate
Acute **** HC
Acute **** LTCS
d
Glycolysis/Gluconeogenesis
P (FDR)
Acute/HC $1.6 \cdot 10^{-8}$
Acute/LTCS $4.8 \cdot 10^{-6}$
Acute ** HC
Acute **** LTCS
Acute **** HC
Acute **** LTCS
Lactate
Pyruvate
Glucose
Acute ** HC
Acute **** LTCS
Acetyl-CoA
Fructose-6-phosphate
Glucose-6-phosphate
Acetate
Acute ns HC
Acute ns LTCS
e
Glycine, serine and threonine metabolism
P (FDR)
Acute/HC $1.7 \cdot 10^{-5}$
Acute/LTCS $5.1 \cdot 10^{-6}$
Choline
Metabolism of glycerophoslipids
Betaine aldehyde
Betaine
Pyruvate
Acute ** HC
Acute **** LTCS
Dimethylglycine
Serine
Sarcosine
Glycine
Acute * HC
Acute * LTCS
Threonine
Creatine
Guanidino-acetate
Acute **** LTCS
f
Arginine and proline metabolism
P (FDR)
Acute/HC $8.8 \cdot 10^{-5}$
Acute/LTCS $1.4 \cdot 10^{-5}$
Guanidino-acetate
Arginine
Peptides
Acute ns HC
Acute ** LTCS
Proline
Ornithine
1-Pyrroline-5-carboxylate
Glutamate-γ-semialdehyde
Creatine
Acute **** HC
Acute **** LTCS
Glutamate
Acute ns HC
Acute ns LTCS

Suppl. Fig. 3

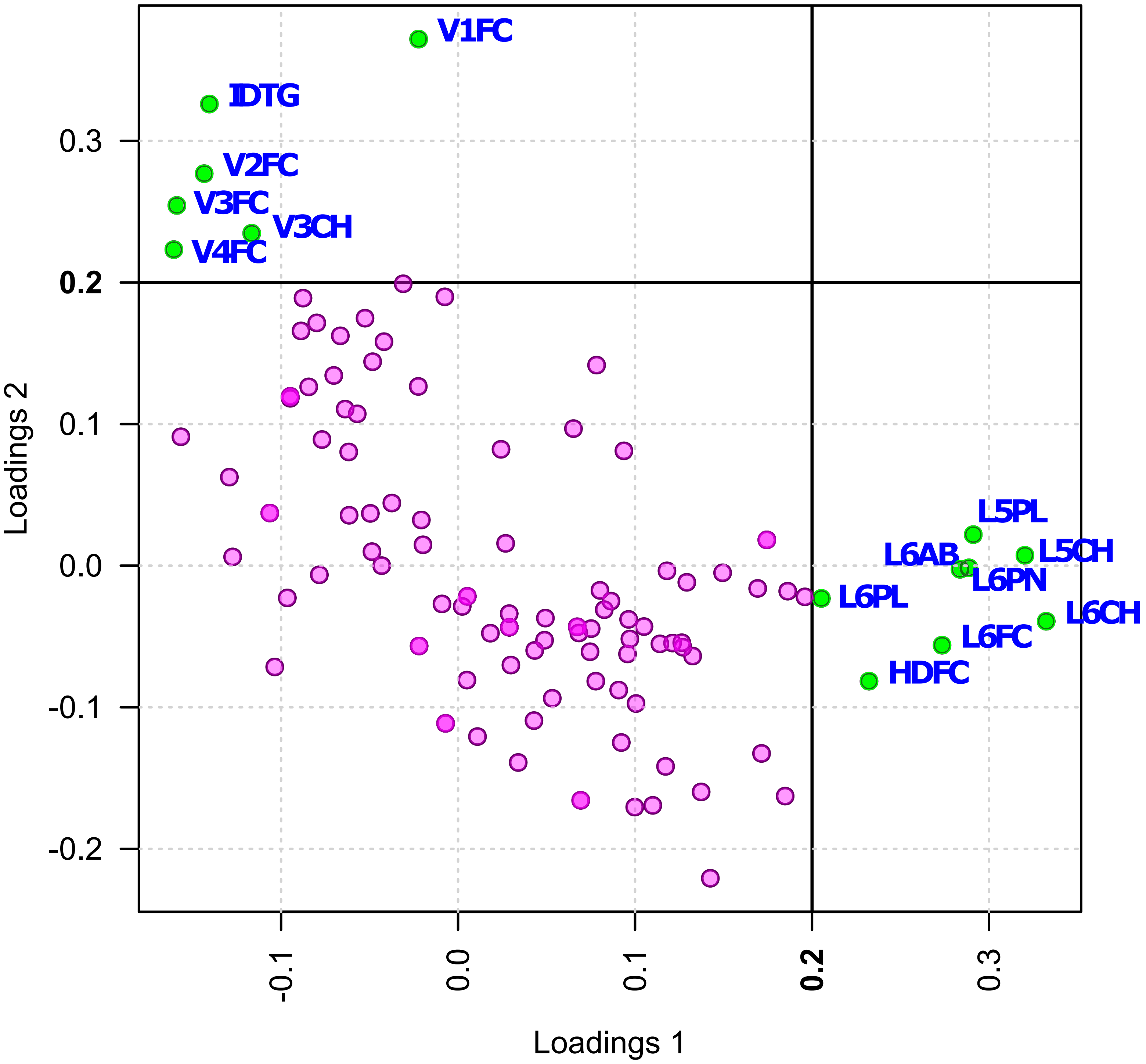
V1FC
IDTG
V2FC
V3FC
V3CH
V4FC
L5PL
L6AB
L5CH
L6PN
L6PL
L6CH
L6FC
HDFC
0.3
0.2
0.1
0.0
-0.1
-0.2
Loadings 2
Loadings 1

Suppl. Fig. 4

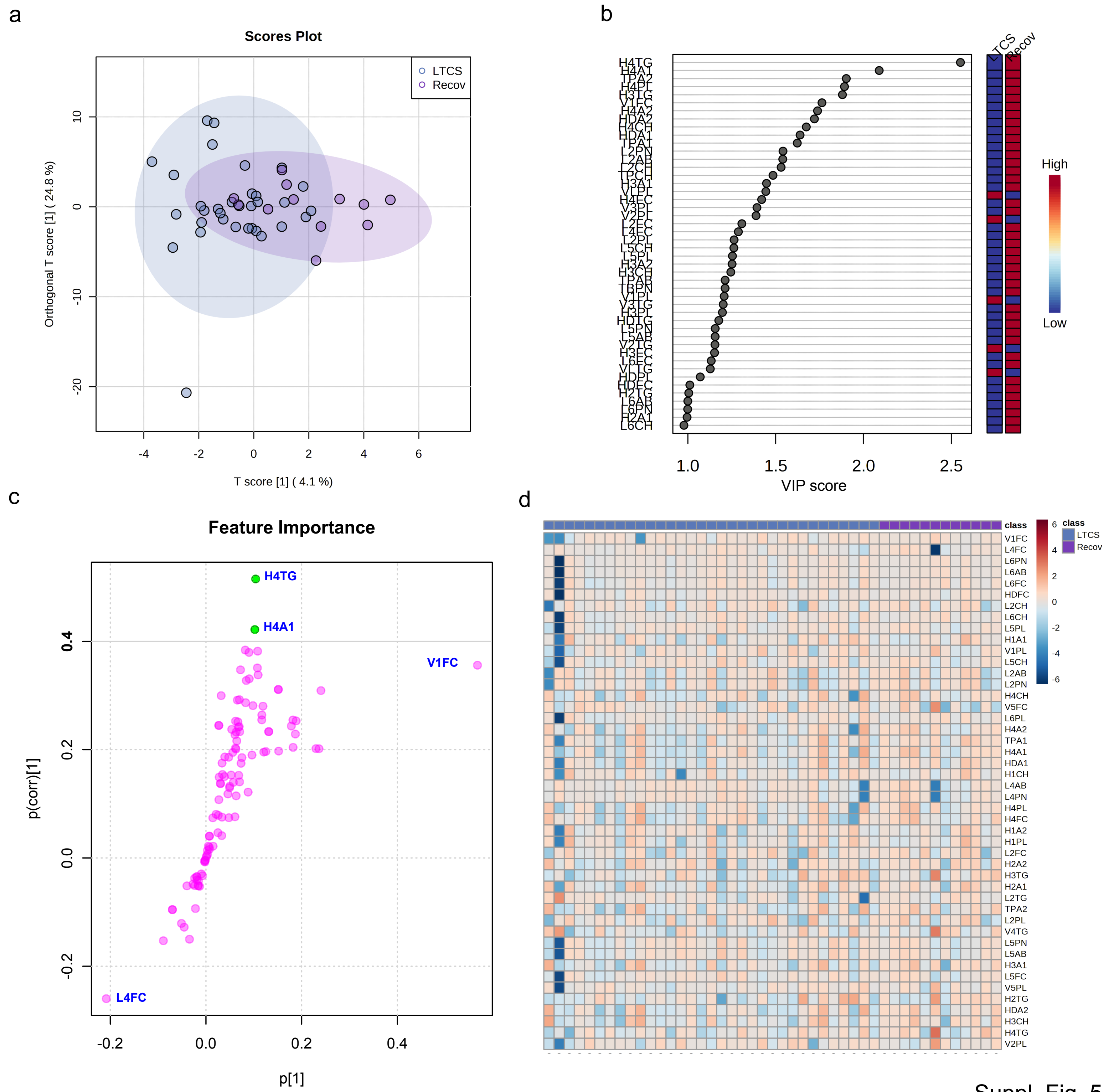
a
Scores Plot
LTCS
Recov
Orthogonal T score [1] (24.8 %)
T score [1] (4.1 %)
b
VIP score
LTCS
Recov
High
Low
H4TG
H4A1
c
Feature Importance
H4TG
H4A1
V1FC
L4FC
p(corr)[1]
p[1]
d
class
LTCS
Recov
V1FC
L4FC
L6PN
L6AB
L6FC
HDFC
L2CH
L6CH
L5PL
H1A1
V1PL
L5CH
L2AB
L2PN
H4CH
V5FC
L6PL
H4A2
TPA1
H4A1
HDA1
H1CH
L4AB
L4PN
H4PL
H4FC
H1A2
H1PL
L2FC
H2A2
H3TG
H2A1
L2TG
TPA2
L2PL
V4TG
L5PN
L5AB
H3A1
L5FC
V5PL
H2TG
HDA2
H3CH
H4TG
V2PL

Suppl. Fig. 5

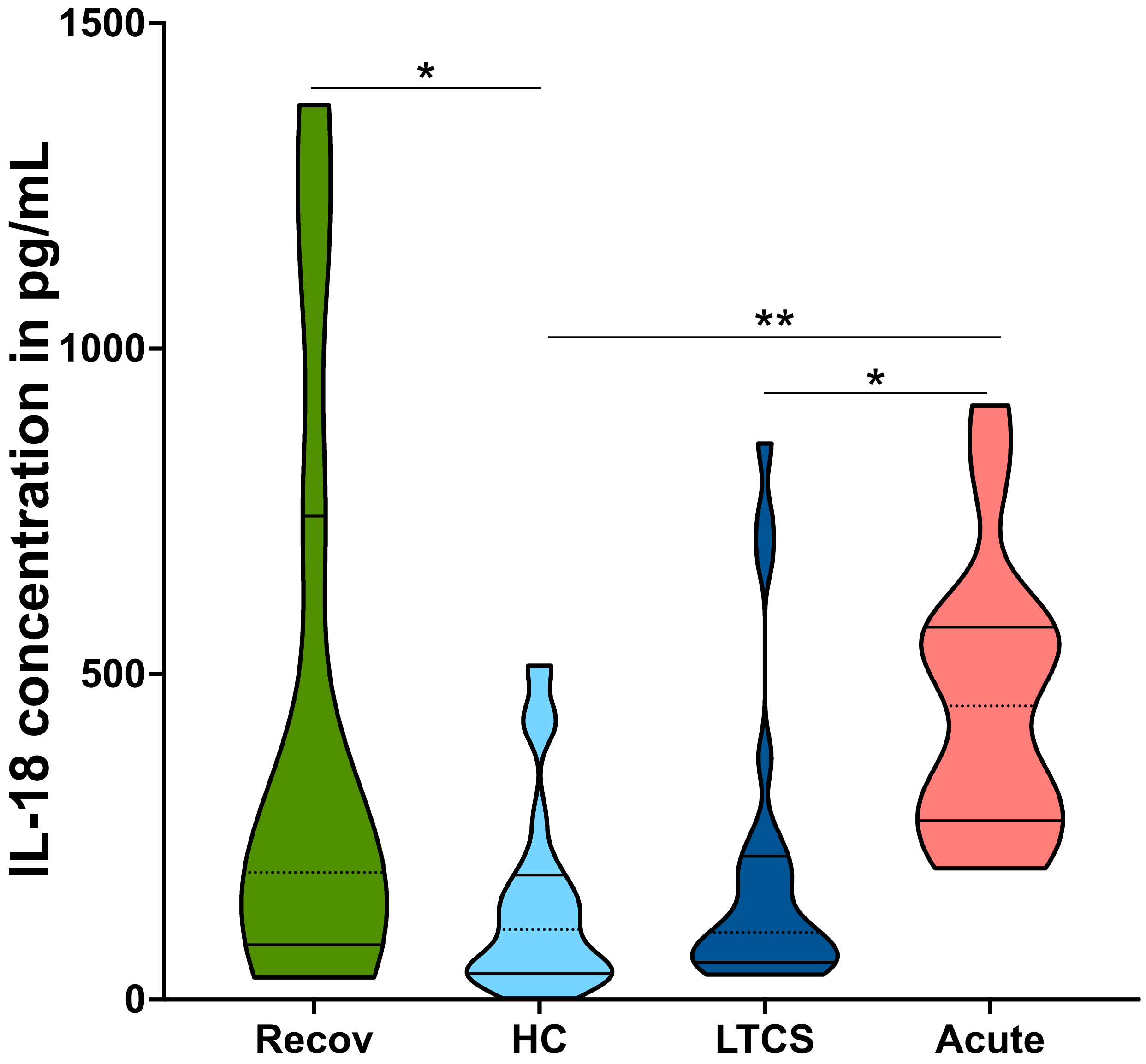
1500
1000
500
0
IL-18 concentration in pg/mL
*
**
*
Recov
HC
LTCS
Acute

Suppl. Fig. 6

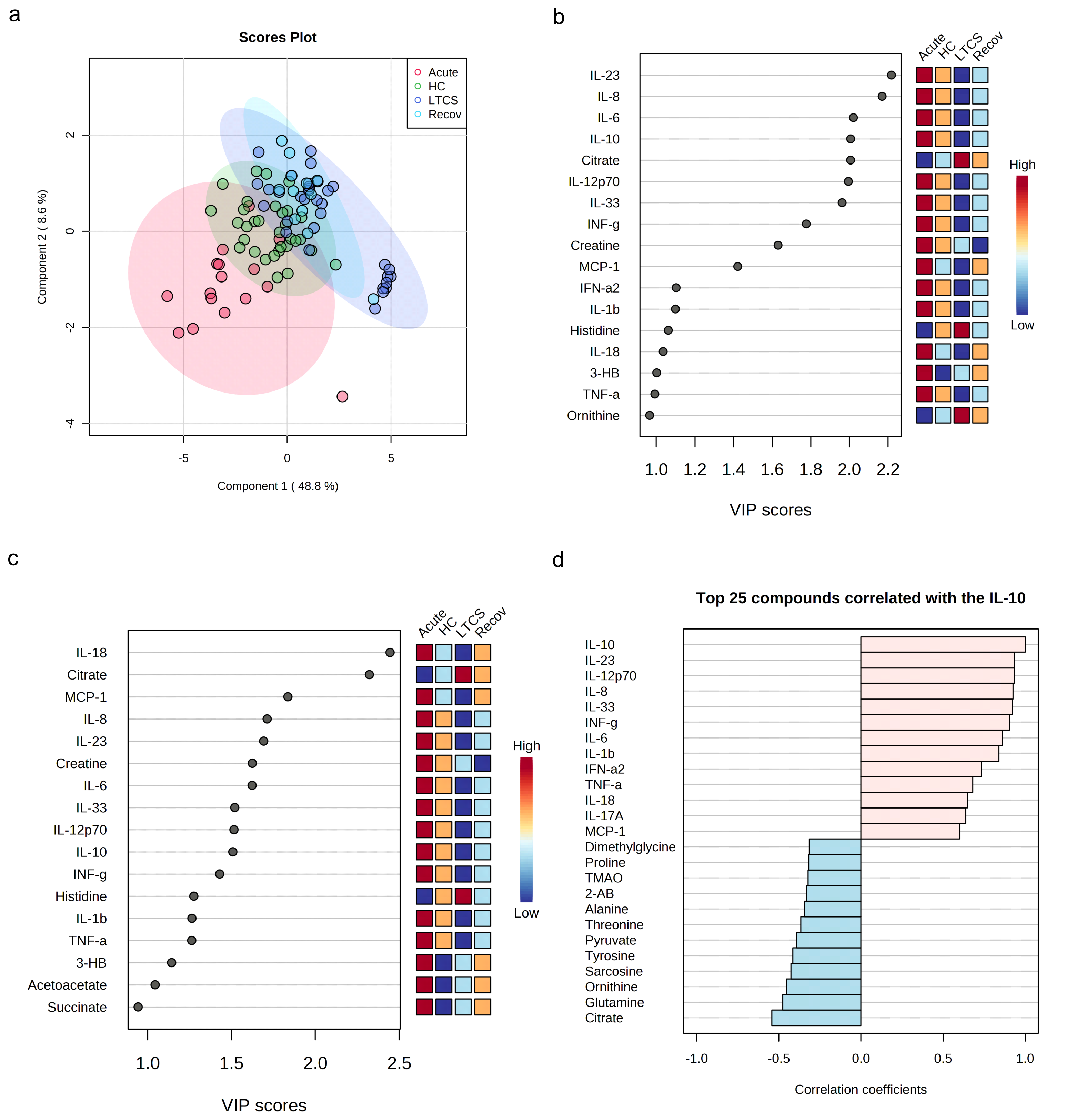
a
Scores Plot
Acute
HC
LTCS
Recov
Component 1 (48.8 %)
Component 2 (8.6 %)
b
IL-23
IL-8
IL-6
IL-10
Citrate
IL-12p70
IL-33
INF-g
Creatine
MCP-1
IFN-a2
IL-1b
Histidine
IL-18
3-HB
TNF-a
Ornithine
High
Low
VIP scores
c
IL-18
Citrate
MCP-1
IL-8
IL-23
Creatine
IL-6
IL-33
IL-12p70
IL-10
INF-g
Histidine
IL-1b
TNF-a
3-HB
Acetoacetate
Succinate
VIP scores
d
Top 25 compounds correlated with the IL-10
IL-10
IL-23
IL-12p70
IL-8
IL-33
INF-g
IL-6
IL-1b
IFN-a2
TNF-a
IL-18
IL-17A
MCP-1
Dimethylglycine
Proline
TMAO
2-AB
Alanine
Threonine
Pyruvate
Tyrosine
Sarcosine
Ornithine
Glutamine
Citrate
Correlation coefficients

Suppl. Fig. 7
